# Haematological traits co-vary with migratory status, altitude and energy expenditure: a phylogenetic, comparative analysis

**DOI:** 10.1038/s41598-019-42921-4

**Published:** 2019-04-23

**Authors:** Kang Nian Yap, Olivia Hsin-I Tsai, Tony D. Williams

**Affiliations:** Department of Biological Sciences, Simon Fraser University, 8888 University Drive, Burnaby, British Columbia, V5A 1S6 Canada

**Keywords:** Ecophysiology, Animal physiology

## Abstract

Aerobic capacity is assumed to be a main predictor of workload ability and haematocrit (Hct) and haemoglobin (Hb) have been suggested as key determinants of aerobic performance. Intraspecific studies have reported increases in Hct and Hb in response to increased workload. Furthermore, Hct and Hb vary markedly among individuals and throughout the annual cycle in free-living birds and it has been suggested that this variation reflects adaptive modulation of these traits to meet seasonal changes in energy demands. We used a comparative dataset of haematological traits, measures of metabolic rate (57 species), and life-history traits (160 species) to test several hypotheses for adaptive variation in haematology in relation to migration and altitude. We then extended these general ideas to test relationships between Hct and basal metabolic rate, daily energy expenditure and activity energy expenditure, using the 57 species that we have metabolic rate information for. We found that at the interspecific level, full migrants have higher Hct and Hb than partial migrants and non-migrants, and that altitude is positively correlated with Hb but not Hct. Hct is positively associated with activity energy expenditure (energy spent specifically on costly activities), suggesting that haematological traits could be adaptively modulated based on life-history traits and that Hct is a potential physiological mediator of energetic constraint.

## Introduction

Whole-organism aerobic capacity is assumed to be one of the main predictors of endurance, or the ability to sustain a high workload in a wide range of animals^1,2^, and haematocrit (Hct) and haemoglobin (Hb) concentration have been proposed as key determinants of aerobic or metabolic performance through their role in oxygen transport and delivery^3–6^. Increases in Hct and Hb in response to increased workload (i.e. exercise training) have been reported in mice^7^, lizards^8^, and fish^9^ (reviewed in Yap *et al*.^10^). Many studies in birds have shown that Hct, and to some extent Hb, increase in response to experimentally increased flight costs^11^, thermogenic demands^12^, or altitude acclimatization^13^. As tissues get more active with increased locomotion and physical activity, their oxygen demand also increases, which in turn triggers higher rate of erythropoiesis resulting in higher Hct and Hb to increase blood oxygen transport capacity^14,15^. Hct and Hb also vary markedly among individuals and through the annual cycle in free-living birds and it has been suggested that this variation reflects adaptive modulation of haematological traits to meet seasonal changes in energy demands, e.g. during migration^3,16–18^ or winter acclimatization^19,20^. For example, Barve *et al*. and Clemens found that birds that reside at high altitude year round have high Hct and Hb relative to lowland birds, and that Hb is correlated with altitude in both high altitude residents and elevational migrants^19,21^, despite the fact that Hct and Hb are relatively plastic traits. It is also well known that Hct and Hb is  increased prior to and during long distance migration in birds^10,16,22^. Furthermore, Hct and Hb are also highly repeatable within individuals and within species^23,24^. From a life-history point of view, in some environmental contexts (e.g. during breeding or migratory seasons), individuals with higher Hct and Hb could “perform better”, e.g. rearing chicks or migrating to breeding grounds faster, and thus will have higher reproductive success and fitness^25^. In other words, the ability and capacity to modulate Hct and Hb could be key adaptations for high-intensity workload associated with different life-history stages^26^.

More generally, although Hct and Hb are clearly only two components of the complex machinery underpinning energy metabolism in animals (other components include mitochondrial function, lung capacity, Hb isoform, muscle physiology, etc.)^3^, they might be functionally significant in determining how animals manage and allocate energy to cope with environmental challenges^27–32^. For instance, when exposed to cold environment, a bird with high Hct and Hb would be able to utilize more energy for shivering thermogenesis, as a result of its higher thermogenic capacity^12^, compared to a bird with low Hct and Hb. Other physiological traits underpinning energy metabolism are often hard to obtain or not available for most species, and hence, only Hct and Hb are chosen for the present study. Common measures of energy expenditure include basal or resting metabolic rate (BMR: the minimum energy required for self-maintenance), field metabolic rate (FMR: the total energy expenditure of an unrestrained animal over the course of 24 h), and activity energy expenditure (AEE: the amount of energy available to fuel behavior, i.e. FMR − BMR)^27,28^. Recently, Mathot & Dingemanse and Portugal *et al*. summarized three different models of energy management^27,28^:

1. The ‘performance’ model, which assumes that the capacity to expend energy at a high rate during activity requires greater maintenance costs (e.g. long distance flight requires maintenance of big flight muscles), and that higher BMR is predicted to be positively related to FMR with a slope > 1.
2. The ‘allocation’ model, which assumes that there is an energetic ceiling, above which animals would suffer from increased risk of mortality due to physical fatigue, predation or infection^33^. This model sets FMR as a fixed amount, thus, it does not vary with BMR, while AEE decreases with increasing BMR. In this case, the predicted slope of FMR-BMR is zero.
3. The ‘independent’ model assumes that there is no energetic ceiling and that the relationship between BMR and AEE are uncoupled. In other words, BMR and AEE can be adjusted independently. However, since BMR is still a component of FMR, higher BMR is predicted to be positively related to FMR, albeit with a slope < 1.

From these models, they suggested that AEE might be a more valid proxy for energetic constraints (i.e. the total amount of energy that can be expended and/or assimilated during a given period of time) (Portugal *et al*., but see Careau and Garland; Mathot and Dingemanse)^28,34,35^.

However, although progress has been made in terms of understanding the relationship between BMR, AEE and FMR^28^, surprisingly little is known about the physiological basis of the observed relationship, as well as the physiology underpinning energetic constraints in general^27^. Fair *et al*. concluded that there are conflicting data concerning changes in Hct in relation to energy expenditure, with studies reporting increases, no change, and decreases in Hct with increased energy expenditure^36^. Similarly, a review by Minias suggested that Hb is positively correlated with a suite of fitness related traits that might reflect energetic constraints, such as egg size and developmental stability, as well as life-history stages associated with high energy demand (e.g. migration), but the relationships were not always consistent between species^37^. Nevertheless, Hct and Hb could still be functionally important in determining how animals manage their energy budget. For instance, animals with high Hct and Hb will likely have high aerobic capacity and therefore, would be able to sustain high intensity exercise and increased activity for longer periods of time, which would in turn potentially result in higher energy expenditure.

To our knowledge there has been no comprehensive, phylogenetically-controlled test of hypotheses for inter-specific variation in hematological traits (Hct and Hb) or the relationship with energy expenditure in general. Furthermore, while there has been a number of studies investigating scaling relationships between body mass and traits like energy expenditure^30,38–40^, organ sizes^41^, and mitochondrial volume^42^ just to name a few, no studies to date have investigated how Hct and Hb scale with body mass at the interspecific level. Fair *et al*. and Minias reviewed sources of variation in Hct and Hb in birds and found that these traits either increased or were not affected by altitude, and that the relationship between Hct and energy expenditure was inconsistent^36,37^. They also discussed how Hct and Hb vary with body mass and attributed the changes observed in hematological traits to variation in body condition^36,37^, rather than a scaling relationship per se. However, neither review used a phylogenetic framework. Therefore, we took a comparative, phylogenetic approach to rigorously test several hypotheses for adaptive variation in Hct and Hb. Specifically, based on findings from previous studies (summarized by Fair *et al*. and Minias)^36,37^, we hypothesized that (1) Hct and Hb would scale positively with body mass, (2) migratory birds will have the highest Hct and Hb levels, followed by partially migratory birds and non-migratory birds, and (3) birds found in higher altitude will have higher Hct and Hb levels than birds found in lower altitude. We then extended these general ideas to test relationships between Hct and BMR, FMR and AEE, in the context of different models of energy management. If Hct is indeed a physiological mediator of energetic constraints more generally^27,28^, we would predict that regardless of life-history traits, Hct will be positively associated with AEE but not associated with FMR and BMR.

Given that Hct and Hb are both relatively plastic traits and can vary depending on environmental conditions, one possible limitation to this study is that measurements of Hct/Hb are often collected during a short and specific time period of the year, which would limit our ability to generalize the findings to other time periods of the year. Unfortunately, despite our best effort to extract sampling date information from individual studies, this is how far we can get with existing datasets from the literature and therefore, readers should keep the limitation in mind while reading the rest of the article.

## Materials and Methods

### Data collection

Between the year 2013 and 2015, the literature (Web of Science and Google Scholar) was surveyed for studies reporting energetics, body mass and haematology, using the search terms ‘basal metabolic rate’, ‘field metabolic rate’, ‘daily energy expenditure’, ‘body mass’, ‘haemoglobin’ and ‘haematocrit’. Searches was conducted over the entire time period. Initial search returned about 400 results, which we then filtered and extracted mean values for Hct, Hb, BMR, FMR, as well as body mass, migratory status and altitude if they were available. Additional Hct and Hb data were obtained from “The Avian Erythrocyte: Its Phylogenetic Odyssey”^43^. Information on migratory status and mean altitude (across all known sites visited year round) were obtained either from the same study, or from the “Birds of North America”^44^, Penguin World^45^, and Bird Life International^46^. Mean altitude is estimated for Important Bird and Biodiversity Areas (IBAs) for a particular species when data is not available; sites selected based on knowledge of “presence and abundance of species that occur there, year round or seasonally”^46^. In total, 137 references were used to compile the dataset for this study (Tables S4 and S5). The season(s) during which birds were sampled were noted whenever possible (Table S4). A total of 160 species were used in the analysis of the relationship between life-history traits (migratory status and altitude) and haematology and a total of 57 species were used in the analysis of energetics (BMR, FMR, AEE) and Hct, with a mean sample size of 15 individuals per species (range 1–265). Hct and Hb measurements were collected from birds in different geographical locations, habitats, and different time of the year. However, due to latitudinal differences in climate and seasonality, as well as the potential auto-correlation and confounding effects of habitat on migratory status, we decided to disregard habitat and include all bird species to maximize our sample sizes for the purpose of this analysis. Body mass was either obtained from the same study, or from the “CRC book of Avian Body Masses”^47^. The studies included in our comparative dataset are comprised of both clinical studies as well as eco-physiological studies, although only non-experimentally manipulated adult populations were considered. When only one reference per species is used, we calculated the mean Hct and/or Hb values of all individuals in that study, and when more than one reference was found for a given species, the values provided in those references were averaged. Since Hct and Hb are highly correlated between males and females, data for different sexes were pooled. In all studies, BMR was measured using flow-through respirometry within the animals’ thermoneutral zone, whereas FMR was either measured using doubly-labelled water technique or estimated using time-energy budget method (it has been demonstrated that both techniques yield reasonable and consistent results)^48^. In all cases, Hct was measured using microhaematocrit centrifugation and Hb was measured using the cyanomethemoglobin method. Units were converted to grams (g) for body mass, percentage for Hct, and Watts (W) for BMR and FMR. AEE was calculated by subtracting BMR from FMR. Data and references for haematology and energetics are provided in tables in Supplementary Information (Tables S4 and S5).

### Phylogenetic Tree Construction

Using the website BirdTree.org, we obtained 500 phylogenetic trees of all species considered in both sets of analyses using the Ericson Sequenced Species backbone posterior distribution from a global phylogeny of birds^49^. Briefly, the tree construction approach combines relaxed clock molecular trees of well-supported avian clades with a fossil calibrated backbone with representatives from each clade. To obtain the phylogenetic trees for the species considered in our analyses, the global phylogeny was first trimmed to a subset, and 500 trees were randomly sampled from a chosen pseudo-posterior distribution (see Jetz *et al*. for detailed methods)^49^. The consensus trees for both sets of analyses are provided in electronic Supplementary Material (S1 and S2).

### Statistical analyses

Analyses were carried out using R version 0.99.467 (R Core Team 2013) and the packages “ape”, “geiger”, “nlme”, “phytools” and “visreg”. Data were first examined for normality using Shapiro-Wilk test. Body mass, Hb, BMR, FMR and AEE were log transformed, whereas data for mean altitude was square-root transformed prior to analysis to improve their distributions. Linear regressions were computed using phylogenetic generalized least squares (PGLS) in which the residuals are modeled as having evolved via a Brownian Motion process^50^. PGLS was conducted in lieu of an ordinary least squares model (OLS) because OLS assumes that each independent data point contributes equally to the estimation of the regression line, whereas PGLS ‘downweights’ points in proportion to the degree of shared phylogenetic history^50^.

We first tested whether Hct and Hb scale with body mass by including body mass as a predictor of Hct and Hb in our regression models. We also tested whether Hct and Hb are correlated at the interspecific level by using Hct as a predictor and Hb as a response variable in our PGLS regression model. To test the hypotheses for adaptive variation in Hct and Hb, altitude was included in the second regression models first as a predictor of Hct and Hb, and subsequently as a covariate along with body mass, when testing for the relationships between migratory status and Hct and Hb. To investigate how Hb varies with migratory status independent of Hct, we also ran a separate regression model using migratory status as a predictor and altitude, body mass, and Hct and covariates. Additionally, for species where we have sampling date information for, and were only sampled during one season, a regression model was run using sampling season as a predictor and altitude, migratory status, and body mass as covariates. Tukey’s HSD (package multcomp)^51^ was used to evaluate pairwise comparisons between migratory status and between sampling seasons following a significant PGLS model.

For energetic traits, in light of previous studies showing positive scaling of body mass to BMR and FMR^28,30,38^, we tested the effect of body mass on measures of energy expenditure, using body mass as a predictor of BMR, FMR and AEE in our PGLS regression models. To test the hypotheses regarding different models of energy management, we tested the relationship between BMR and FMR, BMR and AEE. Potential collinearity and part-whole correlation^52^ between BMR and FMR is addressed by subtracting BMR from FMR (i.e. AEE), and running a separate model with BMR and AEE. It is also common practice to test the relationships between BMR and FMR, and BMR and AEE when determining energy management models^27,28^. Finally, to test relationships between Hct and energy expenditure, we included Hct as a predictor of BMR, AEE and FMR in our regression models. To account for multiple test correction, likelihood ratio tests were conducted to compare models using only body mass as a predictor of energetics measures and models using Hct as predictor of energetic measures. Due to insufficient data for Hb, we could not rigorously test the relationships between Hb and measures of energy expenditure. Degrees of freedom of the residuals, slope, intercept, R-squared values, and p-values were reported for regressions with continuous predictors, while F- and Z-statistics were reported for regressions with categorical variables. Additionally, estimates of the phylogenetic signal associated with all regressions (Pagel’s λ), which indicates the extent to which closely related species tend to resemble each other, were reported as well^50^. A summary of statistical output showing the main variables and predictors are presented in Table 1. The variables used and all PGLS models as well as the detailed statistical output from each model are provided in a table in electronic Supplementary material (Table S3).

**Table 1:** Summary of statistical output showing all variables and PGLS models.

PGLS model	num *DF*	Residual *DF*	*F*-value	Slope	Intercept	*R* ^2^	*P*-value
**log Hb ~ Hct**	.	158	.	0.007	0.85	0.07	<0.01
**Hct ~ migration** + √ altitude + log mass	2	155	4.95	.	.	.	<0.01
**log Hb ~ migration** + √ altitude + log mass	2	155	4.31	.	.	.	0.015
**Hct ~ √ altitude** + log mass	.	157	.	0.03	49.35	<0.01	0.22
**log Hb ~ √ altitude** + log mass	.	157	.	0.001	2.76	0.09	<0.01
**Hct ~ log mass**	.	158	.	−1.57	49.59	<0.01	0.22
**log Hb ~ log mass**	.	158	.	−0.02	1.20	0.02	0.24
**log FMR ~ log mass**	.	56	.	0.62	−1.93	0.64	<0.01
**log BMR ~ log mass**	.	56	.	0.67	−3.33	0.73	<0.01
**log AEE ~ log mass**	.	56	.	0.56	−2.11	0.37	<0.01
**BMR ~ AEE**	.	56	.	0.04	−2.36	0.37	0.85
**BMR ~ FMR**	.	56	.	0.40	−0.81	0.69	<0.01
**log BMR ~ Hct** + log mass	.	54	.	−0.02	−2.40	0.09	0.06
**log FMR ~ Hct** + log mass	.	54	.	0.02	−3.21	<0.01	0.05
**log AEE ~ Hct** + log mass	.	54	.	0.06	−5.28	0.05	<0.01

Main response variables and predictors are highlighted in bold.

## Results

### Do migratory status and altitude predict variation in haematocrit and haemoglobin?

Hct and Hb were significantly positively associated across species (*df* = 158, y = 0.007x + 0.85, p < 0.001, *R*^2^ = 0.07, Pagel’s *λ* = 0.81), but both Hct (*df* = 158, y = −1.53x + 49.90, p = 0.29, R^2^ < 0.01, Pagel’s *λ* = 0.62) and Hb (*df* = 158, y = −0.01x + 1.23, p = 0.40, *R*^2^ = 0.02, Pagel’s *λ* = 0.79) were independent of body mass.

**Figure 1:**
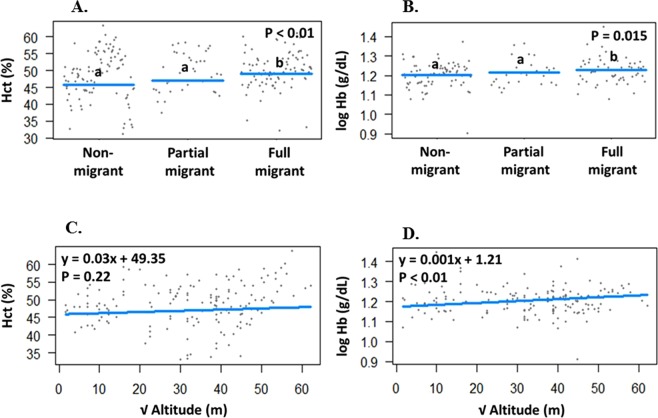
Relationship between (**A**) Hct and migratory status, (**B**) Hb and migratory status, (**C**) Hct and altitude, and (**D**) Hb and altitude. Data shown in 1A and 1B are individual species data and means. Different letters denote statistical significance. Data shown in 1C and 1D are individual species data and PGLS regression line.

There was a significant association between migratory status and Hct (*F*_2,155_ = 4.95, p = 0.007, Pagel’s *λ* = 0.62, Fig. 1A), where full migrants have significantly higher Hct than both partial migrants (tukey, *Z* = 2.79, p = 0.01) and non-migrants (tukey, Z = 2.65, p = 0.02). A similar pattern was found between migratory status and Hb (*F*_2, 155_ = 4.31, p = 0.015, Pagel’s *λ* = 0.82, Fig. 1B), where full migrants have significantly higher Hb than both partial migrants (tukey, Z = 4.31, p < 0.01) and non-migrants (tukey, Z = 2.33, p = 0.05). The relationship between migratory status and Hb holds true even after Hct was included in the regression model as a covariate (*F*_2,154_ = 12.80, p < 0.0001, Pagel’s *λ* = 0.83). Contrary to our initial prediction, Hct was independent of altitude (*df* = 157, y = 0.03x + 49.35, p = 0.22, *R*^2^ < 0.01, Pagel’s *λ* = 0.67, Fig. 1C). However, a significant positive relationship was found between altitude and Hb (*df* = 157, y = 0.001x + 1.21, Pagel’s *λ* = 0.82, *R*^2^ = 0.09, p = 0.005, Fig. 1D). The overall PGLS model showed a significant association between Hct and sampling season (*F*_3,41_ = 26.21, p < 0.001, Pagel’s *λ* = 0.05, Fig. S6). Post-hoc pairwise comparisons showed that birds sampled in the winter had higher Hct than birds sampled in the spring (tukey, Z = 3.87, p < 0.001) and birds sampled in the summer (tukey, Z = 8.19, p < 0.001). Hb was independent of sampling season (*F*_3,41_ = 0.34, p = 0.79, Pagel’s *λ* = 0.09, Fig. S6).

### Does variation in haematocrit predict variation in basal metabolic rate, field metabolic rate and activity energy expenditure?

PGLS indicated a significant positive association between body mass and FMR (*df* = 55, y = 0.62x − 1.93, p < 0.001, *R*^2^ = 0.64, Pagel’s *λ* = 0.58), between body mass and BMR (*df* = 55, y = 0.67x − 3.33, p < 0.001, *R*^2^ = 0.73, Pagel’s *λ* = 0.001), and between body mass and AEE (*df* = 55, y = 0.56x − 2.11, p < 0.001, *R*^2^ = 0.37, Pagel’s *λ* = 0.61). There was a significant positive relationship between BMR and AEE (*df* = 54, y = 3.22x − 1.17, p < 0.001, *R*^2^ = 0.57, Pagel’s *λ* = 0.06), and between BMR and FMR (*df* = 54, y = 3.13x + 0.89, p < 0.001, *R*^2^ = 0.72, Pagel’s *λ* = 0.06).

**Figure 2:**
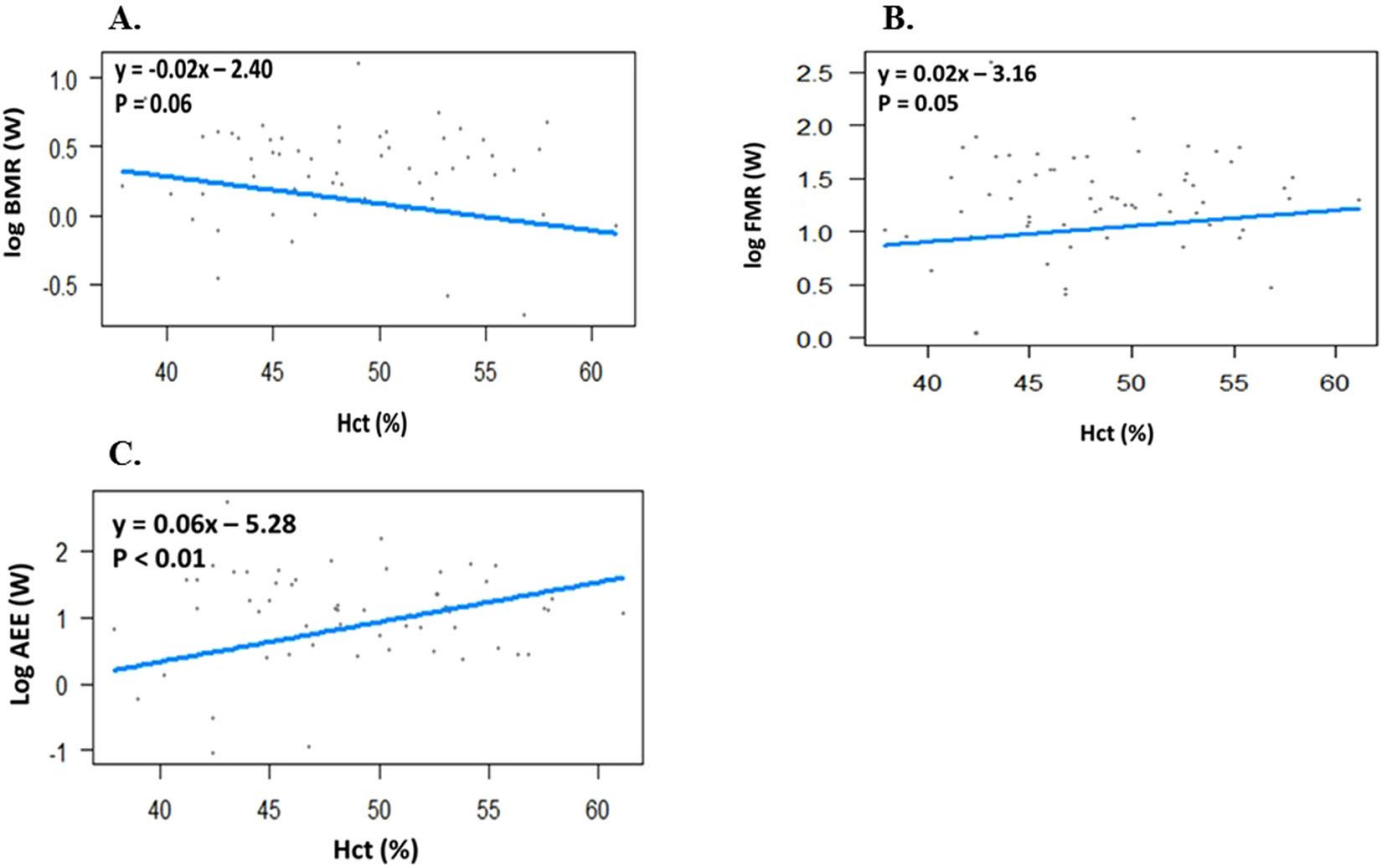
Relationship between (**A**) Hct and BMR, (**B**) Hct and FMR, and (**C**) Hct and AEE. Data shown in 2A-C are individual species data and PGLS regression line.

A non-significant relationship was found between Hct and BMR (*df* = 54, y = −0.02x − 2.40, p = 0.06, *R*^2^ = 0.09, Pagel’s *λ* = 0.13, Fig. 2A) and a marginally significant positive relationship was found between Hct and FMR (*df* = 54, y = 0.02x − 3.16, p = 0.05, *R*^2^ < 0.01, Pagel’s *λ* = 0.58, Fig. 2B). In contrast, a stronger positive relationship was found between Hct and AEE (*df* = 54, y = 0.06x − 5.28, p < 0.001, *R*^2^ = 0.05, Pagel’s *λ* = 0.70, Fig. 2C). Likelihood ratio tests indicated that when predicting interspecific variation in BMR, model was marginally significantly improved when Hct was compared to model based on body mass alone (Chisq = 0.63, p = 0.05). When predicting interspecific variation in FMR and AEE, models were significantly improved when Hct was incorporated into models based on body mass alone (FMR: Chisq = 4.22, p = 0.04; AEE: Chisq = 12.80, p < 0.001).

## Discussion

We took a comparative, phylogenetic approach to rigorously test the hypotheses that (1) Hct and Hb would scale positively with body mass, (2) migratory birds will have the highest Hct and Hb levels, followed by partially migratory birds and non-migratory birds, and (3) birds found in higher altitude will have higher Hct and Hb levels than birds found in lower altitude. With the exception of the regressions for body mass and BMR and for BMR and FMR, most PGLS regressions indicated moderate to strong phylogenetic signal (i.e. Pagels’ *λ* of 0.5 to 0.9). PGLS was used in all of our regressions because it can explicitly take into account phylogenetic signal and control for it appropriately^50^. Consistent with findings from intraspecific studies in endothermic animals (i.e. birds and mammals)^43^, we showed that Hct and Hb are positively correlated at the interspecific level. Perhaps rather surprisingly, contrary to findings from intrapecific studies^36,37^, Hct and Hb do not scale with body mass at the interspecific level. This discrepancy in findings is probably due to the fact that hematology and body mass are not directly related, and that the positive associations seen at the intraspecific level are mostly mediated by body condition of individuals (i.e. healthier individuals having higher body mass and higher Hct/Hb). We also showed that full migrants have higher Hct and Hb than partial migrants and non-migrants, largely consistent with common assumptions in the literature^36,37^. This result is also consistent with previous intraspecific studies looking at partial migrants^53^, where some individuals of the population that migrate have either increased erythropoiesis or Hct, whereas the others that stay as residents maintain low levels of erythropoiesis and consequently low Hct^16,18^. Interestingly, the relationship between migratory status and Hb holds true even after Hct has been included as a covariate, suggesting that migrants do not only have more erythrocytes per unit of blood in their circulatory system, but they also have higher mean cell haemoglobin content. Contrary to our initial prediction, birds found at higher altitude do not have higher Hct than birds found at lower altitude but they do have higher Hb. Increases in Hct and Hb as a means to increase blood oxygen carrying capacity is a well-documented acclimatization response to hypoxia in many vertebrates^13,21,36,54–56^. However, an increase in Hct also results in an exponential increase in viscosity, thus hindering blood oxygen transport^57,58^. By having high mean cell Hb concentration and relatively low Hct, animals can have low blood viscosity without compromising oxygen carrying capacity. This is consistent with findings from Barve *et al*., who found that birds with different migration patterns (e.g. elevational migrants vs. residents) appear to adopt alternative physiological strategies to regulate blood oxygen carrying capacity, suggesting that this phenomenon is perhaps an adaptation for animals that experience fluctuating oxygen demand regularly^21^.

Before attempting to investigate role of Hct as a potential physiological basis of interspecific variation in energy expenditure, we sought to test how different measures of energy expenditures are related to each other, as well as to variation in body masses. Interspecific variation in energy expenditure was partly explained by variation in body mass: all measures of energy expenditure (BMR, AEE and FMR) scale positively with body mass, consistent with findings from other studies^28,29,31,32,38^. In terms of how different measures of energy expenditure relate to each other, our study showed that there were positive correlations between BMR and AEE, and BMR and FMR with a slope > 1. This is contrary to findings of other intraspecific studies that looked at associations between measures of energy expenditure, which found that most bird species employ the independent model of energy management^28,29^. Our study indicated that birds tend to employ the performance model of energy management. In other words, it appears that variation in basic energy requirements (BMR) predicts the capacity to expend energy at high rate during activity (AEE), as well as the total energy expenditure (FMR). The coupled relationship between BMR and AEE makes biological sense when we consider the fact that the capacity to expend energy at a high rate during activity requires greater maintenance costs. However, one should be cautious about distinguishing among energy management models based on the relationship between BMR and FMR alone since trade-offs can occur at either the among- or within-species level^35^.

We sought to investigate the potential physiological basis of interspecific variation in energy expenditure and tested the relationships between Hct and BMR, FMR and AEE. In support of our initial prediction, we found that Hct is positively and more strongly related to AEE than to BMR and FMR at the interspecific level (based on slope of regression). As mentioned before, AEE, as opposed to BMR and FMR, has been suggested to be a more valid proxy for energetic constraints as it is a measure of how much energy can be spent specifically on energetically costly activities^27,28^. Although, many studies have found positive relationships between body mass and measures of energy expenditure^28,29,59,60^, few studies have explored the physiology underpinning energetics. To the best of our knowledge, our study is one of the first to explore the physiological basis of energetic constraints from an interspecific perspective. Our findings that interspecific variation in AEE can be explained by variation in Hct, and that variation in Hct can help explain some of the residual variation in the relationships between body mass and measures of energy expenditure, suggested that perhaps Hct is a mediator of energetic constraint. Knowing that hematological traits and energetics vary across seasons, and between sexes and age, it should be noted that different studies compiled in our dataset likely sampled different individuals at different time of the year and therefore, there might be a strong masking effect of co-variates which have not been controlled for in the study design (but see next paragraph). It should also be noted that some of the significant models (e.g. altitude and Hb, Hct and AEE) had relatively small *R*^2^ values and the slope of the regressions are relatively shallow, suggesting that perhaps there is very little biological relevance despite the observed statistical significance. However, we need to be cautious about interpreting *R*^2^ values obtained from PGLS since they are not comparable with *R*^2^ values obtained from ordinary least squares (OLS) models. Residuals calculated from PGLS are not orthogonal and therefore, it is difficult to ascribe portions of the explained variation to independent variables^50,61^. In light of this finding, future studies should look at how manipulation of Hct can affect the way animals allocate and manage energy.

As we mentioned earlier, we acknowledge that there are a number of limitations in our study, some of which were related to logistical constraints, e.g. sample sizes included in our compiled studies, time of the year when birds were sampled, sampling populations included in the compiled studies, etc. For example, migratory birds are sometimes only sampled during the migratory seasons when Hct and Hb are high. Therefore, it limits our ability to generalize the findings to other time periods of the year. However, based on the data for species where we have sampling date information for, birds sampled during the spring and fall seasons (i.e. migratory season) did not seem to have higher Hct and Hb compared to birds sampled during the other seasons, suggesting that the high Hct and Hb values observed in migratory species are likely to be a real biological phenomenon. The observation that birds sampled in the winter had higher Hct than birds sampled in the summer and spring may just be an artifact of low sample sizes. Out of the five species with the highest Hct values, only *Plegadis falcinellus* and *Catharacta maccormicki* are migratory species, and aside from *Catharacta maccormicki*, most other birds are found predominantly in warm tropical climate.

In summary, our study has shown that interspecific variation in Hct and Hb can be explained by altitude and migratory status of birds, and that Hct is a potential physiological mediator of energetic constraints and trade-offs in birds. We know that there are interspecific variations in reproductive effort and output in animals^62–65^, both of which are key determinants of Darwinian fitness and life-history trade-offs. Given the potential role of Hct in mediating energetic constraints, it remains to be determined if interspecific variation in reproductive effort and output such as clutch size and egg size can be explained by variation in Hct.

## Supplementary information

Supplementary information and dataset

## Data Availability

All data analyzed during this study are included in this published article (and its Supplementary Information files- Figures S1, S2 and Tables S3–S5).
