# Supplementary material for: Haematological traits co-vary with migratory status, altitude and energy expenditure: a phylogenetic, comparative analysis
Source: Sci Rep. 2019 Apr 23;9:6351. doi: 10.1038/s41598-019-42921-4 (PMC6476874; doi:10.1038/s41598-019-42921-4)

Haematological traits co-vary with migratory status, altitude and energy expenditure: a phylogenetic, comparative analysis

Kang Nian Yap, Olivia Hsin-I Tsai, Tony D. Williams

Supplementary materials

Figure S1. Consensus tree for analyses of the relationship between life-history traits and haematology.

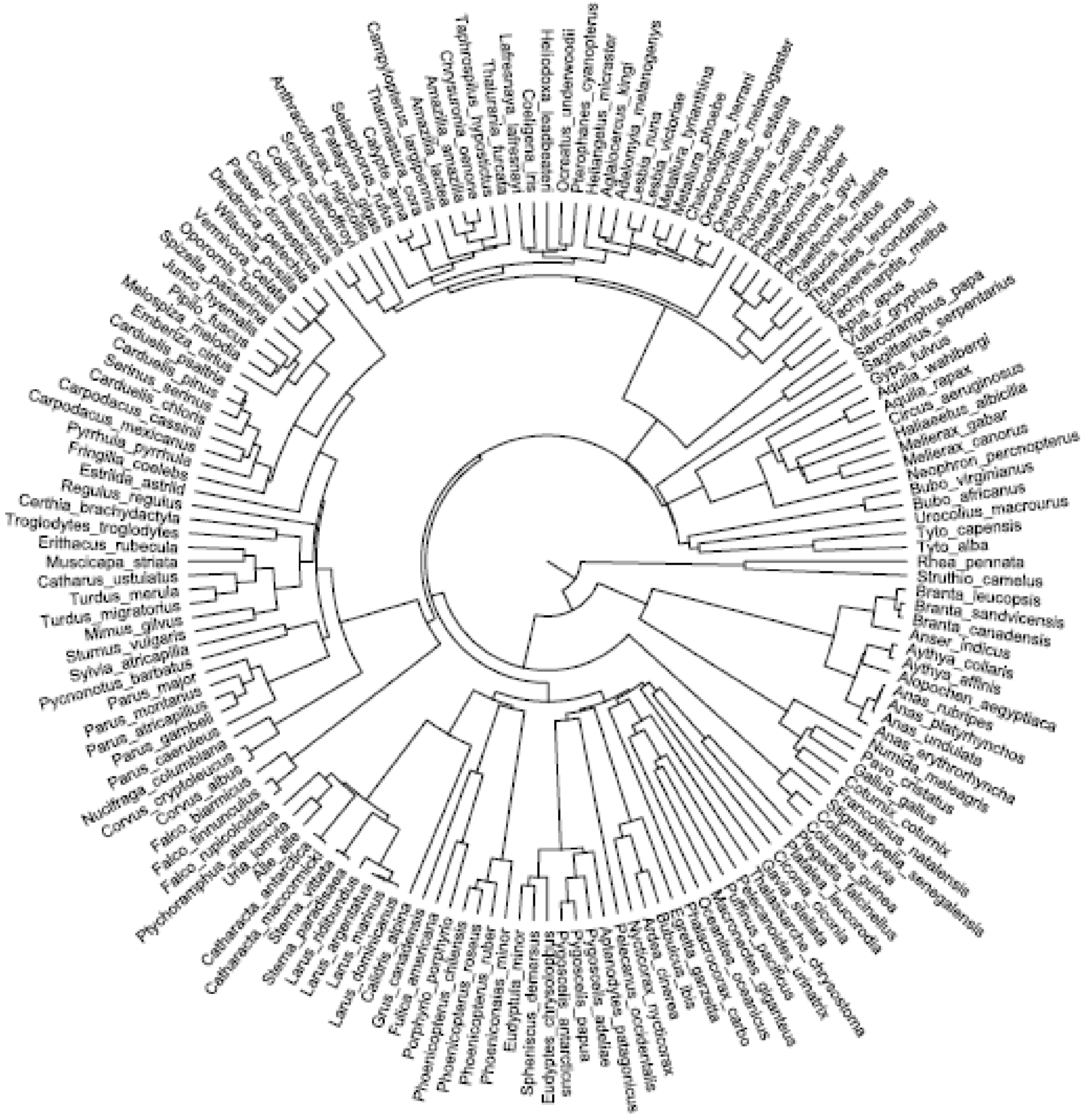

Figure S2. Consensus tree for analyses of the relationship between haematocrit and measures of energy expenditure.

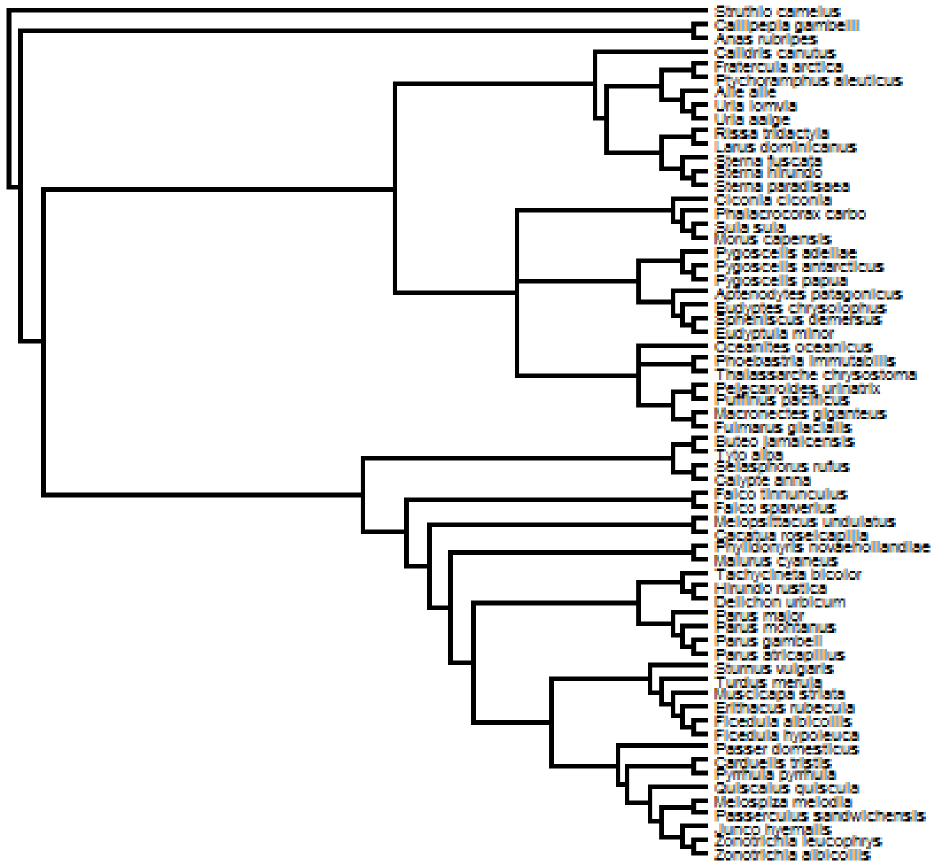

Table S3. Statistical output showing all variables and PGLS models. Response variables are highlighted in bold. Corresponding predictors are underlined.

PGLS model	num*DF*	Residual *DF*	*F*-value	Slope	Intercept	R^2	*P*-value
**log Hb** ~ Hct	.	158	.	0.007	0.85	0.07	< 0.01
**Hct** ~ migration + √ altitude + log mass	2	155	4.95	.	.	.	< 0.01
**Hct** ~ migration + √ altitude + log mass	.	155	.	0.01	49.59	.	0.76
**Hct** ~ migration + √ altitude + log mass	.	155	.	-0.759	49.59	.	0.22
**Hct** ~ season + √ altitude + log mass	3	41	26.21	.	.	.	< 0.01
**log Hb** ~ migration + √ altitude + log mass	2	155	4.31	.	.	.	0.015
**log Hb** ~ migration + √ altitude + log mass	.	155	.	0.001	2.822	.	0.445
**log Hb** ~ migration + √ altitude + log mass	.	155	.	-0.012	2.822	.	0.525
**log Hb** ~ migration + √ altitude + Hct + log mass	2	154	12.80	.	.	.	< 0.01
**log Hb** ~ migration + √ altitude + Hct + log mass	.	154	.	0.003	2.668	.	0.525
**log Hb** ~ migration + √ altitude + Hct + log mass	.	154	.	0.002	2.668	.	0.432
**log Hb** ~ migration + √ altitude + Hct + log mass	.	154	.	-0.008	2.668	.	0.673
**log Hb** ~ season + √ altitude + log mass	3	41	0.35	.	.	.	0.792
**Hct** ~ √ altitude + log mass	.	157	.	0.03	49.35	< 0.01	0.220
**Hct** ~ √ altitude + log mass	.	157	.	0.301	49.35	.	0.252
**log Hb** ~ √ altitude + log mass	.	157	.	0.002	2.764	0.09	< 0.01
**log Hb** ~ √ altitude + log mass	.	157	.	-0.009	2.764	.	0.646
**Hct** ~ log mass	.	158	.	-1.57	49.59	< 0.01	0.22
**log Hb** ~ log mass	.	158	.	-0.02	1.20	0.02	0.24
**log FMR** ~ log mass	.	56	.	0.62	-1.93	0.64	< 0.01
**log BMR** ~ log mass	.	56	.	0.67	-3.33	0.73	< 0.01
**log AEE** ~ log mass	.	56	.	0.56	-2.11	0.37	< 0.01
**BMR ~ AEE**	.	56	.	0.04	-2.36	0.37	0.85
**BMR ~ FMR**	.	56	.	0.40	-0.81	0.69	< 0.01
**log BMR** ~ Hct + log mass	.	54	.	-0.02	-2.40	0.09	0.06
**log BMR** ~ Hct + log mass	.	54	.	0.66	-2.40	.	<0.01
**log FMR** ~ Hct + log mass	.	54	.	0.02	-3.21	< 0.01	0.05
**log FMR** ~ Hct + log mass		54	.	0.64	-3.21	.	<0.01
**log AEE** ~ Hct + log mass	.	54	.	0.06	-5.28	0.05	< 0.01
**log AEE** ~ Hct + log mass		54	.	0.61	-5.28	.	<0.01

Table S4. Data and references for haematology and life-history traits.

Species	Migration	Altitude (m)	Mass (g)	Hct (%)	Hb (g/dL)	Hematology Sample size	Sampling Season	Hematology Ref.
Calidris alpina	yes	2.5	52.5	47.3	11.06	22	spring	(Verhulst, Oosterbeek, & Bruinzeel, 2002)
Phoenicopterus ruber	yes	2.5	3096.5	46	15.4	15	Not applicable (tropical)	(Peinado, Polo, Viscor, & Palomeque', 1992)
Phoeniconaias minor	no	4	1500	45	15.4	2	Not applicable (tropical)	(Peinado et al., 1992)
Pygoscelis papua	no	14	5848	45.3	16.8	20	summer	(Cranfield, 2003; Glomski & Pica, 2011; Hawkey & Dennett, 1989; Milsom, Johansen, & Millard, 1973; Myrcha & Kostelecka-Myrcha, 1980; Myres et al., 1972)
Larus dominicanus	no	15	980.3	39	12.6	21	summer	(Glomski & Pica, 2011; Myrcha & Kostelecka-Myrcha, 1980; Myres et al., 1972)
Phoenicopterus roseus	yes	21.5	3035	49	18	15	Not applicable (tropical)	(Peinado et al., 1992)
Catharacta maccormicki	yes	25	1349	46.4	13.87	1	winter	(Ibañez, Najle, Larsen, & Montalti, 2015)
Larus marinus	partial	29	800	41	12.9	50	winter	(Averbeck, 1992; Glomski & Pica, 2011)
Sterna vittata	yes	50	159.6	47.9	14.4	20	summer	(Myres et al., 1972)
Puffinus pacificus	yes	50	384	48	14.8	9	unspecified	(Glomski & Pica, 2011; Work, 1996)
Sterna paradisaea	yes	50	101.125	46	14.4	40	unspecified	(A Kostelecka-Myrcha, Jaroszewicz, & Cholostiakow-Gromek, 1993)
Eudyptula minor	no	50	1120.25	41.2	13.8	20	unspecified	(Nicol, Melrose, & Stahel, 1988)
Aptenodytes patagonicus	yes	50	13500	47.8	16.7	35	unspecified	(Glomski & Pica, 2011; Speckmann, 1986)
Ptychoramphus aleuticus	partial	50	173.989	53	20.7	12	spring	(Elliott, Shoji, Campbell, & Gaston, 2010)
Uria lomvia	partial	58	980	52.8	18	8	summer	(Croll, Gaston, Burger, & Konnoff, 1992;

								Dunphy et al., 2015)
Pelecanoides urinatrix	yes	68	137	42.4	17.6	21	fall	(Dunphy et al., 2015)
Anas rubripes	partial	75	1026	40.2	13	1	unspecified	(Glomski & Pica, 2011; Ritchie,B. W., Hsarrison, G. J., Zantop, D., & Harrison, 1994)
Platalea leucorodia	yes	88	1950	46.4	15.9	24	winter	(Celdrán, Polo, Peinado, Viscor, & Palomeque, 1994)
Phalacrocorax carbo	yes	98	1950	45.4	14.55	13	unspecified	(Balasch, Palomeque, Palacios, Musquera, & Jimenez, 1974)
Spheniscus demersus	no	100	3169	46.7	15.4	41	unspecified	(Glomski & Pica, 2011; Speckmann, 1986)
Thalassarche chrysostoma	yes	100	3706.25	41.67	16.06	15	summer	(Crossin, Phillips, Wynne-Edwards, & Williams, 2013)
Eudyptes chrysolophus	yes	100	3870	50.35	24.36	48	spring	(Crossin et al., 2010)
Grus canadensis	partial	112	4300	42.75	13.8	56	fall	(Gee, Carpenter, & Hensler, 1981)
Pygoscelis adeliae	yes	125	3970	46.2	16.5	24	summer	(Myres et al., 1972)
Catharacta antarctica	yes	139	1401	42.2	12.7	10	summer	(Myres et al., 1972)
Haliaeetus albicilla	no	142	4033.44	45	15.3	5	unspecified	(Glomski & Pica, 2011)
Macronectes giganteus	yes	150	3939.0625	43.4	14.6	14	summer	(Glomski & Pica, 2011; Milsom et al., 1973)
Pelecanus occidentalis	yes	150	3510	44.5	15.3	3	unspecified	(Carpenter, 1975)
Larus ridibundus	yes	150.5	214.9	43.12	14.5	12	unspecified	(Balasch et al., 1974)
Aythya collaris	yes	177	705	49.1	14.3	2	unspecified	(Glomski & Pica, 2011; Kocan, 1972)
Branta leucopsis	yes	200	2100	43.1	11.26	5	unspecified	(Lague, Chua, Farrell, Wang, & Milsom, 2016)
Fringilla coelebs	partial	202.5	22.5	49.5	17.3	17	spring, summer	(Glomski & Pica, 2011; A Kostelecka-Myrcha et al., 1993; Palomeque, Palacios, & Planas, 1980; Palomeque & Planas, 1981)
Campylopterus largipennis	no	250	8.97	61	20.23	4	unspecified	(Gregory, Andrews, McGuire, & Witt, 2009;

								Parker, Douglas F. Stotz, & John W. Fitzpatrick, 1996)
Plegadis falcinellus	yes	250	650	49.9	17.1	12	winter	(Celdrán et al., 1994)
Gavia stellata	yes	250	1255	54	20.7	1	unspecified	(Bond & Gilbert, 1958)
Pygoscelis antarcticus	yes	250	3515.5	50.1	19.6	21	summer	(Myres et al., 1972)
Aythya affinis	yes	294.5	820	57.25	16	30	unspecified	(Kocan, 1972)
Alle alle	yes	400	1644.267	56.8	17	26	unspecified	(Glomski & Pica, 2011; Alina Kostelecka-Myrcha, 1987)
Florisuga mellivora	no	450	7.16	56	18.65	4	unspecified	(Gregory et al., 2009; Parker et al., 1996)
Emberiza cirlus	no	450	20	39	14.2	1	spring	(Palomeque, Palacios, et al., 1980)
Estrilda astrild	no	500	6.9	44.3	14.94	10	spring	(Girard & Grima, 1980)
Anthracothorax nigricollis	yes	500	7.92	51	18.4	1	unspecified	(Gregory et al., 2009; Parker et al., 1996)
Anas platyrhynchos	partial	500	1000	38.2	13.91	12	spring, summer, fall	(Driver, 1981; Glomski & Pica, 2011; Olayemi & Arowolo, 2009)
Coturnix coturnix	yes	500	96.63	41.208	14.56	14	unspecified	(Prats, Palacios, Gallego, & Riera, 1996)
Serinus serinus	yes	525	9	50	17.7	1	spring	(Palomeque, Palacios, et al., 1980)
Glaucis hirsutus	no	550	6.53	56	18.5	4	unspecified	(Gregory et al., 2009; Parker et al., 1996)
Phaethornis hispidus	no	600	5.28	54	18.17	3	unspecified	(Gregory et al., 2009; Parker et al., 1996)
Amazilia lactea	no	700	5.19	59	19.95	1	unspecified	(Gregory et al., 2009; Parker et al., 1996)
Certhia brachydactyla	no	711	7	44	15.9	1	spring	(Palomeque, Palacios, et al., 1980)
Vermivora celata	yes	750	9.26	54.63	17.7	11	spring, fall	(Bennett, G. F., 1964; Carey & Morton, 1976)
Amazilia amazilia	no	750	5.15	51	16.63	4	unspecified	(Gregory et al., 2009; Johansen, K., Berger, M., Bicudo, J. E. P. W., Ruschi, A., 1983; Parker et al., 1996)
Phaethornis ruber	no	750	3.72	47	15.6	4	Not applicable (tropical)	(Peinado et al., 1992)
Sarcoramphus papa	no	750	3400	42.7	14.4	6	unspecified	(Balasch, Musquera,

								Palacios, Jimenez, & Palomeque, 1976)
Rhea pennata	no	750	23900	48	17.8	36	fall	(Reissig, Robles, & Sager, 2002)
Threnetes leucurus	no	800	6.36	52	18.15	1	unspecified	(Gregory et al., 2009; Parker et al., 1996)
Phaethornis malaris	no	825	6.11	53	17.2	4	unspecified	(Gregory et al., 2009; Parker et al., 1996)
Chrysuronia oenone	no	825	4.53	53	17.95	4	unspecified	(Gregory et al., 2009; Parker et al., 1996)
Thalurania furcata	no	850	5.13	54	17.83	4	unspecified	(Gregory et al., 2009; Parker et al., 1996)
Taphrospilus hypostictus	no	875	6.83	55	18.3	1	unspecified	(Gregory et al., 2009; Parker et al., 1996)
Calypte anna	yes	900	4.3	56.3	18.2	1	spring, fall	(Carey & Morton, 1976)
Francolinus natalensis	no	900	516	31.75	11.4	24	unspecified	(Le & Hattingh, 1983)
Egretta garzetta	partial	1000	515	50	12.4	12	winter	(Celdrán et al., 1994; Glomski & Pica, 2011)
Gallus gallus	no	1000	1673.6	47.8	13.63	83	unspecified	(Aengwanich, 2007; Hammond, Chappell, Cardullo, Lin, & Johnsen, 2000)
Erithacus rubecula	yes	1000	16.3	53.45	15.32	34	spring, summer, fall, winter	(Norte et al., 2013)
Branta canadensis	partial	1000	1940	42	13.2	27	unspecified	(Gee et al., 1981)
Pavo cristatus	no	1000	3903	36.15	12.2	10	unspecified	(Balasch et al., 1973)
Larus argentatus	yes	1000	1085	43.2	15.9	12	unspecified	(Balasch et al., 1974)
Sturnus vulgaris	partial	1000	73.9	57.5	23	5	summer	(Van den Steen et al., 2010)
Melierax gabar	no	1000	177.5	32.8	16.4	2	unspecified	(Cooper, 1975)
Melierax canorus	no	1000	675	32.8	16.4	1	unspecified	(Cooper, 1975)
Urocolius macrourus	no	1050	51.3	54.5	18.8	31	winter	(Prinzinger & Misovic, 1994)
Bubo africanus	no	1050	645	33.17	12.42	8	unspecified	(Le & Hattingh, 1983)
Falco rupicoloides	no	1075	261	44.25	14.14	6	unspecified	(Le & Hattingh, 1983)
Muscicapa striata	yes	1100	14.4	55.433	15.84	2	summer	(Bennett, G. F., 1964)
Sylvia atricapilla	partial	1100	21.9	46	16.25	8	spring	(Glomski & Pica, 2011; A Kostelecka-Myrcha et al., 1993; Palomeque, Palacios, et al., 1980)

Turdus merula	partial	1150	82.6	45	15.9	3	spring	(Palomeque, Palacios, et al., 1980)
Carduelis chloris	yes	1179.5	20.5	45	14.6	2	spring	(Palomeque, Palacios, et al., 1980)
Thaumastura cora	no	1200	2.43	58	18.45	1	unspecified	(Gregory et al., 2009; Parker et al., 1996)
Mimus gilvus	no	1250	62	47	12.89	12	unspecified	(Gálvez, Carlos Fernando; Ramírez, Ginés Fernando; Osorio, 2010)
Porphyrio porphyrio	no	1250	919.3	47.6	15.9	10	winter	(Celdrán et al., 1994; Glomski & Pica, 2011)
Melospiza melodia	partial	1400	21.6	52.6	17	25	spring, fall	(Carey & Morton, 1976; Glomski & Pica, 2011)
Heliodoxa leadbeateri	no	1400	6.74	51	17.22	5	unspecified	(Gregory et al., 2009; Parker et al., 1996)
Tachymarptis melba	yes	1400	85.45	51.05	18.65	5	summer	(Palomeque, Rodriguez, Palacios, & Planas, 1980)
Aquila wahlbergi	yes	1400	640	32.5	16.9	2	unspecified	(Cooper, 1975)
Oceanites oceanicus	yes	1483	42.692	49.3	17	76	summer	(Glomski & Pica, 2011; Myrcha & Kostelecka - Myrcha, 1980; Myres et al., 1972)
Dendroica petechia	yes	1500	9.11	50.2	13.5	2	spring, fall	(Carey & Morton, 1976)
Circus aeruginosus	yes	1500	711.5	49.31	14.31	13	unspecified	(Lavin, Cuenca, Marco, Velarde, & Viñas, 1992)
Numida meleagris	no	1500	1520	45.5	13.61	15	unspecified	(Balasch et al., 1973; Glomski & Pica, 2011; Le & Hattingh, 1983; Le R. Fourie & Hattingh, 1980)
Catharus ustulatus	yes	1500	27	58.4	18	1	spring, fall	(Carey & Morton, 1976)
Anas erythrorhyncha	no	1500	559.5	44.58	14	16	unspecified	(Le & Hattingh, 1983)
Pycnonotus barbatus	no	1500	40.3	45	14.3	4	unspecified	(Glomski & Pica, 2011; Le & Hattingh, 1983)
Phaethornis guy	no	1500	5.09	55	17.55	4	unspecified	(Gregory et al., 2009; Parker et al., 1996)
Gyps fulvus	yes	1500	7600	44.6	14.55	32	winter	(Balasch et al., 1976; Glomski & Pica, 2011; Palomeque & Planas, 1977; Polo, Celdrán, Peinado, Viscor,

								& Palomeque, 1992)
Carpodacus mexicanus	no	1500	19.56	50.8	18.4	3	spring, fall	(Carey & Morton, 1976)
Stigmatopelia senegalensis	partial	1500	108	37.6	13.8	4	unspecified	(Le & Hattingh, 1983)
Struthio camelus	no	1500	88300	45	16.9	135	unspecified	(Levi et al., 1989)
Columba guinea	no	1500	352	49.88	19.45	4	unspecified	(Le & Hattingh, 1983)
Aquila rapax	no	1500	2250	34	13.79	3	unspecified	(Le & Hattingh, 1983)
Carduelis psaltria	no	1550	9.6	52.5	17	1	spring, fall	(Carey & Morton, 1976)
Pipilo fuscus	no	1550	43.99	44.1	15.1	5	spring, fall	(Carey & Morton, 1976)
Parus atricapillus	no	1600	10.4	48.1	12.6	16	spring, fall	(Bennett, 1986; Carey & Morton, 1976; Glomski & Pica, 2011)
Tyto capensis	no	1600	419	39.6	12.71	10	unspecified	(Le & Hattingh, 1983)
Apus apus	yes	1650	40.5	50	17.7	23	summer	(Glomski & Pica, 2011; Palomeque, Palacios, et al., 1980)
Sagittarius serpentarius	no	1660	3607	37.5	22.3	2	unspecified	(Cooper, 1975)
Schistes geoffroyi	no	1700	3.91	59	19.28	4	unspecified	(Gregory et al., 2009; Parker et al., 1996)
Fulica americana	yes	1700	550	46	17	2	unspecified	(Bond & Gilbert, 1958)
Eutoxeres condamini	no	1740	10.73	52	16.85	2	unspecified	(Gregory et al., 2009; Parker et al., 1996)
Wilsonia pusilla	yes	1750	7.2	54.53	16.7	14	summer	(Bennett, G. F., 1964)
Adelomyia melanogenys	no	1750	3.1	58	18.03	4	unspecified	(Gregory et al., 2009; Parker et al., 1996)
Ciconia ciconia	yes	1750	3440	44.1	13.9	7	spring	(Alonso et al., 1991)
Parus caeruleus	no	1750	9.7	39	13.7	1	summer	(Palomeque, Palacios, et al., 1980)
Falco biarmicus	no	1750	616	46	16.52	9	unspecified	(Le & Hattingh, 1983)
Falco tinnunculus	partial	1750	213	45.9	17.3	18	unspecified	(Glomski & Pica, 2011; Kirkwood, Cooper, & Brown, 1979)
Pyrrhula pyrrhula	partial	1750	29.5	53.8	20.9	10	spring	(Glomski & Pica, 2011; A Kostelecka-Myrcha et al., 1993)
Aglaiocercus kingi	partial	1800	4.77	50	15.73	2	unspecified	(Gregory et al., 2009; Parker et al., 1996)
Oporornis tolmiei	yes	1850	10.4	58.45	19.4	3	spring, fall	(Carey & Morton, 1976)

Corvus albus	no	1850	520	39	14.39	4	unspecified	(Le & Hattingh, 1983)
Spizella passerina	partial	1900	11.9	53.5	16.75	20	summer	(Bennett, G. F., 1964)
Anas undulata	no	1945	1008	40.67	13.38	17	unspecified	(Le & Hattingh, 1983)
Selasphorus rufus	yes	1967	3.6	61.1	17.7	5	spring, fall	(Carey & Morton, 1976)
Bubo virginianus	no	2000	1588	32	7.7	1	unspecified	(Bond & Gilbert, 1958)
Ardea cinerea	partial	2000	1443	40	13.16	7	unspecified	(Le & Hattingh, 1983)
Branta sandvicensis	no	2000	1970	46	15.5	17	unspecified	(Gee et al., 1981)
Alopochen aegyptiaca	no	2000	1873	43.75	14.95	8	unspecified	(Le & Hattingh, 1983)
Tyto alba	no	2000	533.2	42.42	15.5	10	unspecified	(Bartsch, Ball, Rosenzweig, & Salman, 1937; Cooper, 1975; Ferrer, García-Rodríouez, Carrillo, & Castroviejo, 1987; Glomski & Pica, 2011; Le & Hattingh, 1983)
Regulus regulus	yes	2000	3	51	28.9	19	summer, fall	(Glomski & Pica, 2011; Merila & Svenssont, 2017)
Ocreatus underwoodii	partial	2025	2.82	58	18.43	2	unspecified	(Gregory et al., 2009; Parker et al., 1996)
Bubulcus ibis	yes	2040	366	45	13.7	9	winter	(Celdrán et al., 1994)
Colibri thalassinus	no	2100	6	56	17.6	1	unspecified	(Gregory et al., 2009; Parker et al., 1996)
Parus gambeli	partial	2122.5	11.7	50	15.7	14	summer	(Williams & Braun, 1996)
Parus montanus	no	2137.5	11.5	44.9	15	5	spring	(Glomski & Pica, 2011; A Kostelecka-Myrcha et al., 1993)
Corvus cryptoleucus	yes	2200	480	47.8	14.9	5	unspecified	(Carmi, Pinshow, Horowitz, & Bernstein, 1993)
Parus major	no	2210	17.7	44.5	13.5	42	summer	(Glomski & Pica, 2011; Hauptmanová et al., 2002; Hõrak, Ots, & Murumägi, 1998; Kostelecka-myrcha, 1997; A Kostelecka-Myrcha et al., 1993)

Phoenicopterus chilensis	yes	2250	2277	44.97	14.37	57	unspecified	(Peinado et al., 1992; M. L. Puerta, Huecas, & Del Campo, 1989)
Neophron percnopterus	partial	2250	2082	42.5	13.7	5	fall	(Polo et al., 1992)
Columba livia	no	2250	296.53	50.54	16.68	265	unspecified	(Glomski & Pica, 2011; Kaplan, 1954; Pavlak, Vlahović, Jerčić, Dovč, & Župančić, 2005)
Passer domesticus	no	2250	29.3	48.25	16.95	11	summer, spring	(Bartsch et al., 1937; Bush, F. M., & Townsend, 1971; Glomski & Pica, 2011; A Kostelecka-Myrcha et al., 1993; M. Puerta, Nava, Venero, & Veiga, 1995)
Troglodytes troglodytes	partial	2287.5	8.9	56	22	9	spring	(Glomski & Pica, 2011; A Kostelecka-Myrcha et al., 1993)
Turdus migratorius	yes	2350	79.76	50.34	15.94	37	spring, fall	(Bennett, G. F., 1964; Carey & Morton, 1976)
Nycticorax nycticorax	yes	2400	810	45.4	14	15	winter	(Celdrán et al., 1994)
Anser indicus	yes	2500	2600	47.8	13.9	6	unspecified	(Black & Tenney, 1980; Lague et al., 2016)
Coeligena iris	no	2500	6.46	58	17.9	4	unspecified	(Gregory et al., 2009; Parker et al., 1996)
Vultur gryphus	no	2500	11350	42.85	13.425	11	unspecified	(Balasch et al., 1976; Gee et al., 1981)
Nucifraga columbiana	no	2550	13.16	52.05	15.9	2	spring, fall	(Carey & Morton, 1976)
Polyonymus caroli	no	2550	4.76	61	19	2	unspecified	(Gregory et al., 2009; Parker et al., 1996)
Carpodacus cassinii	yes	2550	25.91	56.05	20.9	7	spring, fall	(Carey & Morton, 1976)
Lafresnaya lafresnayi	partial	2650	5.15	59	19.27	3	unspecified	(Gregory et al., 2009; Parker et al., 1996)
Lesbia nuna	no	2750	3.17	52	18.8	1	unspecified	(Gregory et al., 2009; Parker et al., 1996)
Junco hyemalis	partial	2800	20.8	50.45	15.4	10	spring, fall	(Carey & Morton, 1976; Nice, Nice, & Kraft, 1935)
Metallura tyrianthina	partial	2850	3.64	60	19.18	4	unspecified	(Gregory et al., 2009; Parker et al., 1996)

Heliangelus micraster	no	2850	5.32	58	18.83	4	unspecified	(Gregory et al., 2009; Parker et al., 1996)
Carduelis pinus	partial	2900	13.8	54	18.15	46	summer, winter	(Bennett, G. F., 1964; Carey & Morton, 1976)
Metallura phoebe	no	3000	6.08	61	18.28	3	unspecified	(Gregory et al., 2009; Parker et al., 1996)
Patagona gigas	yes	3000	21.16	55	17.23	4	unspecified	(Gregory et al., 2009; Parker et al., 1996)
Colibri coruscans	no	3100	9	62	19.8	4	unspecified	(Gregory et al., 2009; Parker et al., 1996)
Pterophanes cyanopterus	no	3150	10.6	59	19.2	4	unspecified	(Gregory et al., 2009; Parker et al., 1996)
Lesbia victoriae	no	3300	4.06	66	20.6	1	unspecified	(Gregory et al., 2009; Parker et al., 1996)
Chalcostigma herrani	no	3350	5.75	53	18.55	2	unspecified	(Gregory et al., 2009; Parker et al., 1996)
Oreotrochilus estella	no	3700	8.1	54.9	16.73	4	unspecified	(Carpenter, 1975)
Oreotrochilus melanogaster	no	3850	6.24	56	15.85	4	unspecified	(Gregory et al., 2009; Parker et al., 1996)

References

Aengwanich, W. (2007). Effects of high environmental temperature on blood indices of Thai indigenous chickens, Thai indigenous chickens crossbred and broilers. *International Journal of Poultry Science*, *6*(6), 427–430.

Alonso, J. C., Huecas, V., Alonso, J. A., Abelenda, M., Muñoz-Pulido, R., & Puerta, M. L. (1991). Hematology and blood chemistry of adult White Storks (Ciconia ciconia). *Comparative Biochemistry and Physiology, Part A*, *98*(3–4). doi:10.1016/0300-9629(91)90421-8

Averbeck, C. (1992). Hematology and Blood-Chemistry of Healthy and Clinically Abnormal Great Black-Backed Gulls (Larus-Marinus) and Herring-Gulls (Larus-Argentatus). *Avian Pathology*, *21*(2), 215–223. Retrieved from isi:A1992JA77500002

Balasch, J., Musquera, S., Palacios, L., Jimenez, M., & Palomeque, J. (1976). Comparative hematology of some Falconiforms. *The Condor*, *78*, 258–259.

Balasch, J., Palacios, L., Musquera, S., Palomeque, J., Jimenez, M., & Alemany, M. (1973). Comparative hematological values of several Galliformes. *Poultry Science*, *52*, 1531–1534.

Balasch, J., Palomeque, J., Palacios, L., Musquera, S., & Jimenez, M. (1974). Hematological values of some great flying and aquatic-diving birds. *Comparative Biochemistry and Physiology -- Part A: Physiology*, *49*(1), 137–145. doi:10.1016/0300-9629(74)90549-0

Bartsch, P., Ball, W. H., Rosenzweig, W., & Salman, S. (1937). Size of red corpuscles and their nucleus in fifty North American birds. *Auk*, *54*, 516–519.

Bennett, G. F., and A. E. C. (1964). Measurements on the blood cells of some wild birds of North America. *Wildlife Disease*, *38*, 1–22.

Bennett, P. M. (1986). *Comparative studies in morphology, life history and ecology among birds*.

Bird Life International. (2018).

Black, C. P., & Tenney, S. M. (1980). Oxygen transport during progressive hypoxia in high-altitude and sea-level waterfowl. *Respiration Physiology*, *39*(2), 217–239. doi:10.1016/0034-5687(80)90046-8

Bond, C. F., & Gilbert, P. W. (1958). Comparative study of blood volume in representative aquatic and nonaquatic birds. *The American Journal of Physiology*, *194*(3), 519–521.

Bush, F. M., & Townsend, J. I. (1971). Ontogeny of hemoglobin in the house sparrow. *Development*, *25*(1), 33–45.

Carey, C., & Morton, M. L. (1976). Aspects of circulatory physiology of montane and lowland birds. *Comparative Biochemistry and Physiology -- Part A: Physiology*, *54*(1), 61–74. doi:10.1016/S0300-9629(76)80073-4

Carmi, N., Pinshow, B., Horowitz, M., & Bernstein, M. H. (1993). Birds conserve plasma volume during thermal and flight-incurred dehydration. *Physiological Zoology*, *66*(5), 829–846.

Carpenter, F. L. (1975). Bird hematocrits: Effects of high altitude and strength of flight. *Comparative Biochemistry and Physiology -- Part A: Physiology*, *50*(2), 415–417. doi:10.1016/0300-9629(75)90035-3

Celdrán, J., Polo, F. J., Peinado, V. I., Viscor, G., & Palomeque, J. (1994). Haematology of captive herons, egrets, spoonbill, ibis and gallinule. *Comparative Biochemistry and Physiology -- Part A: Physiology*, *107*(2), 337–341. doi:10.1016/0300-9629(94)90390-5

Cooper, J. E. (1975). Hematological investigations in east african birds of prey. *Journal of Wildlife Diseases*, *11*(3), 389–394. doi:10.7589/0090-3558-11.3.389

Cranfield, M. R. (2003). Sphenisciformes (penguins). In M. E. Fowler & R. E. Miller (Eds.), *Zoo and Wild Animal Medicine* (5th ed., pp. 103–110). St. Louis, MO: Elsvier Science.

Croll, D. A., Gaston, A. J., Burger, A. E., & Konnoff, D. (1992). Foraging behavior and physiological adaptation for diving in thick- billed murres. *Ecology*. doi:10.2307/1938746

Crossin, G. T., Phillips, R. A., Wynne-Edwards, K. E., & Williams, T. D. (2013). Postmigratory Body Condition and Ovarian Steroid Production Predict Breeding Decisions by Female Gray-Headed Albatrosses. *Physiological and Biochemical Zoology*, *86*(6), 761–768. doi:10.1086/673755

Crossin, G. T., Trathan, P. N., Phillips, R. A., Dawson, A., Le Bouard, F., & Williams, T. D. (2010). A Carryover Effect of Migration Underlies Individual Variation in Reproductive Readiness and Extreme Egg Size Dimorphism in Macaroni Penguins. *The American Naturalist*, *176*(3), 357–366. doi:10.1086/655223

Davis, L. S. (2018). Penguin World. Retrieved from http://www.penguinworld.com/

Driver, E. A. (1981). Hematological and blood chemical values of mallard, Anas p. platyrhynchos, drakes

before, during and after remige moult. *J Wildl Dis*, *17*(3), 413–421. doi:10.7589/0090-3558-17.3.413

Dunning Jr., J. B. (2008). *CRC Handbook of Avian Body Masses*. *CRC handbook of avian body masses. Second edition.* doi:10.1017/S0963180113000479

Dunphy, B. J., Taylor, G. A., Landers, T. J., Sagar, R. L., Chilvers, B. L., Ranjard, L., & Rayner, M. J. (2015). Comparative seabird diving physiology: First measures of haematological parameters and oxygen stores in three New Zealand Procellariiformes. *Marine Ecology Progress Series*, *523*, 187–198. doi:10.3354/meps11195

Elliott, K. H., Shoji, A., Campbell, K. L., & Gaston, A. J. (2010). Oxygen stores and foraging behavior of two sympatric, planktivorous alcids. *Aquatic Biology*, *8*(3), 221–235. doi:10.3354/ab00236

Ferrer, M., García-Rodríouez, T., Carrillo, J. C., & Castroviejo, J. (1987). Hematocrit and blood chemistry values in captive raptors (Gyps Fulvus, Buteo Buteo, Milvus m ig Ra` s, Aquila Heliaca). *Comparative Biochemistry and Physiology -- Part A: Physiology*, *87*(4), 1123–1127. doi:10.1016/0300-9629(87)90049-1

Gálvez, Carlos Fernando; Ramírez, Ginés Fernando; Osorio, J. H. (2010). Parámetros Hematológicos de la Mirla Mimus gilvus (Paseriformes: Mimidae) en cautiverio. *Boletín Científico. Centro de Museos. Museo de Historia Natural*, *14*(26), 120–128.

Gee, G. F., Carpenter, J. W., & Hensler, G. L. (1981). Species Differences in Hematological Values of Captive Cranes, Geese, Raptors, and Quail. *The Journal of Wildlife Management*, *45*(2), 463–483. doi:10.2307/3807928

Girard, H., & Grima, M. (1980). Allometric relation between blood oxygen uptake and body mass in birds. *Comparative Biochemistry and Physiology*, *66*(66), 485–491.

Glomski, C. A., & Pica, A. (2011). *The avian erythrocyte: its phylogenetic odyssey*. CRC PRESS.

Gregory, T. R., Andrews, C. B., McGuire, J. A., & Witt, C. C. (2009). The smallest avian genomes are found in hummingbirds. *Proceedings of the Royal Society B: Biological Sciences*, *276*(1674), 3753–3757. doi:10.1098/rspb.2009.1004

Hammond, K. a, Chappell, M. a, Cardullo, R. a, Lin, R., & Johnsen, T. S. (2000). The mechanistic basis of aerobic performance variation in red junglefowl. *The Journal of Experimental Biology*, *203*(Pt 13), 2053–2064.

Hauptmanová, K., Literák, I., Bártová, E., Hauptmanov, K., Literk, I., Bartova, E., … Bártová, E. (2002). Haematology and Leucocytozoonosis of Great Tits (*Parus major L.*) During Winter. *Acta Veterinaria Brno*, *71*, 199–204. doi:10.2754/avb200271020199

Hawkey, C. M., & Dennett, T. B. (1989). *Color Atlas Of Comparative Veterinary Hematology*. *Veterinary Clinical Pathology* (Vol. 18). Ames, IA: Iowa State University Press. doi:10.1111/j.1939-165X.1989.tb00532.x

Hõrak, P., Ots, I., & Murumägi, A. (1998). Haematological health state indices of reproducing Great Tits: A response to brood size manipulation. *Functional Ecology*, *12*(5), 750–756. doi:10.1046/j.1365-2435.1998.00244.x

Ibañez, A. E., Najle, R., Larsen, K., & Montalti, D. (2015). Hematology, Biochemistry and Serum Protein Analyses of Antarctic and non-Antarctic Skuas. *Waterbirds*, *38*(2), 153–161. doi:10.1675/063.038.0204

Johansen, K., Berger, M., Bicudo, J. E. P. W., Ruschi, A., D. A. (1983). Respiratory properties of blood and myoglobin in hummingbirds. *Physiological Zoology*, *60*(1), 269–78. doi:10.1086/664584

Kaplan, H. M. (1954). Sex differences in the packed cell volume of vertebrate blood. *Science*, *120*(3129), 1044. doi:10.1126/science.120.3129.1044

Kirkwood, J. K., Cooper, J. E., & Brown, G. (1979). Some hematological data for the european kestrel Falco tinnunculus. *Research in Veterinary Science*, *26*(2), 263–264.

Kocan, R. M. (1972). Some physiological blood values of wild diving ducks. *Journal Of Wildlife Diseases*, *8*(8), 115–118.

Kostelecka-myrcha, A. (1997). The ratio of amount of haemoglobin to total surface area of erythrocytes in birds in relation to body mass, age of nestlings, and season of the year. *Physiological Zoology*, *70*(3), 278–282. doi:10.1086/639597

Kostelecka-Myrcha, A. (1987). Respiratory function of a unit of blood volume in the little auk (Plautus alle) and the arctic tern (Sterna paradisaea). *Comparative Biochemistry and Physiology -- Part A: Physiology*, *86*(1), 117–120. doi:10.1016/0300-9629(87)90287-8

Kostelecka-Myrcha, A., Jaroszewicz, M., & Cholostiakow-Gromek, J. (1993). Relationship between the values of red blood indices and the body mass of birds. *Acta Ornithologica*, *28*(1), 47–53.

Lague, S. L., Chua, B., Farrell, A. P., Wang, Y., & Milsom, W. K. (2016). Altitude matters: differences in cardiovascular and respiratory responses to hypoxia in bar-headed geese reared at high and low altitudes. *The Journal of Experimental Biology*, *219*(13), 1974–1984. doi:10.1242/jeb.132431

Lavin, S., Cuenca, R., Marco, I., Velarde, R., & Viñas, L. (1992). Hematology and blood chemistry of the marsh harrier (Circus aeruginosus). *Comparative Biochemistry and Physiology -- Part A: Physiology*, *103*(3), 493–495. doi:10.1016/0300-9629(92)90277-W

Le, F., & Hattingh, J. (1983). Comparative haematology of some South African birds. *Comparative Biochemistry and Physiology -- Part A: Physiology*, *74*(2), 443–448. doi:10.1016/0300-9629(83)90628-X

Le R. Fourie, F., & Hattingh, J. (1980). Variability in bird haematology. *Comparative Biochemistry and Physiology -- Part A: Physiology*, *65*(1), 147–150. doi:10.1016/0300-9629(80)90396-5

Levi, A., Perelman, B., Waner, T., Van Grevenbroek, M., Van Creveld, C., & Yagil, R. (1989). Haematological parameters of the otrich (*Struthio camelus*). *Avian Pathology*, *18*(2), 321–327. doi:10.1080/03079458908418605

Merila, J., & Svenssont, E. (2017). Fat Reserves and Health State in Migrant Goldcrest Regulus regulus Author (s): J . Merila and E . Svensson Published by : British Ecological Society Stable URL : http://www.jstor.org/stable/2389981 JSTOR is a not-for-profit service that helps scholars , *9*(6), 842–848.

Milsom, W. K., Johansen, K., & Millard, R. W. (1973). Blood respiratory properties in some Antarctic birds. *Condor*, *75*(4), 472–474.

Myrcha, A., & Kostelecka - Myrcha, A. (1980). Hematological studies on Antarctic birds I . Hematological indices in some species of the birds studied during australian summer. *Polish Polar Research*, *1*(2–3), 169–173.

Myres, M. T., Myrcha, A., Kostelecka - Myrcha, A., Myres, M. T., Myrcha, A., & Kostelecka - Myrcha, A. (1972). Blood Picture in Some Species of Antarctic Birds. *Bull. Acad. Polon. Sci. Ser. Sci. Biol.*, *27*(11), 911–915.

Nice, L. B., Nice, M. M., & Kraft, R. M. (1935). Erythrocytes and hemoglobin in the blood of some American birds. *The Wilson Bulletin*, *47*, 120–124.

Nicol, S. C., Melrose, W., & Stahel, C. D. (1988). Haematology and metabolism of the blood of the little penguin, Eudyptula minor. *Comparative Biochemistry and Physiology -- Part A: Physiology*, *89*(3), 383–386. doi:10.1016/0300-9629(88)91044-4

Norte, A. C., Lobato, D. N. C., Braga, E. M., Antonini, Y., Lacorte, G., Gonçalves, M., … Ramos, J. A. (2013). Do ticks and Borrelia burgdorferi s.l. constitute a burden to birds? *Parasitology Research*, *112*(5), 1903–1912. doi:10.1007/s00436-013-3343-1

Olayemi, F. O., & Arowolo, R. O. A. (2009). Seasonal variations in the haematological values of the nigerian duck (Anas platyrhynchos). *International Journal of Poultry Science*, *8*(8), 813–815. doi:10.3923/ijps.2009.813.815

Palomeque, J., Palacios, L., & Planas, J. (1980). Comparative respiratory functions of blood in some passeriform birds. *Comparative Biochemistry and Physiology -- Part A: Physiology*, *66*(4), 619–624. doi:10.1016/0300-9629(80)90008-0

Palomeque, J., & Planas, J. (1977). DIMENSIONS OF THE ERYTHROCYTES OF BIRDS. *Ibis*, *119*(4), 533–535. doi:10.1111/j.1474-919X.1977.tb02065.x

Palomeque, J., & Planas, J. (1981). Erythrocyte size in some wild Spanish birds. *Revista Espa¤ola de Fisiolog¡a*, *37*(1), 17–22.

Palomeque, J., Rodriguez, J. D., Palacios, L., & Planas, J. (1980). Blood respiratory properties of swifts. *Comparative Biochemistry and Physiology -- Part A: Physiology*, *67*(1), 91–95. doi:10.1016/0300-9629(80)90412-0

Parker, T. A., Douglas F. Stotz, & John W. Fitzpatrick. (1996). Ecological and Distributional Databases. In *Neotropical birds : ecology and conservation* (pp. 111–410). doi:Cited By (since 1996) 111\rExport Date 12 August 2012

Pavlak, M., Vlahović, K., Jerčić, J., Dovč, A., & Župančić, Ž. (2005). Age, sexual and seasonal differences of haematological values and antibody status to Chlamydophila sp. in feral and racing pigeons (Columba livia forma domestica) from an urban environment (Zagreb, Croatia). *European Journal of Wildlife Research*. doi:10.1007/s10344-005-0110-3

Peinado, V. I., Polo, F. J., Viscor, G., & Palomeque', J. (1992). Haematology and blood chemistry values for several flamingo species. *Avian Pathology*, *21*(1), 55–64. doi:10.1080/03079459208418818

Polo, F. J., Celdrán, J. F., Peinado, V. I., Viscor, G., & Palomeque, J. (1992). Hematological Values for Four Species of Birds of Prey. *The Condor*, *94*, 1007–1013. Retrieved from http://www.jstor.org/stable/1369300

Prats, M. T., Palacios, L., Gallego, S., & Riera, M. (1996). Blood oxygen transport properties during migration to higher altitude of wild quail, Coturnix coturnix coturnix. *Physiological Zoology*, *69*(4), 912–929.

Prinzinger, R., & Misovic, A. (1994). Blood of birds — an allometric review of its components. *Journal of Ornithology*. doi:10.1007/BF01640285

Puerta, M. L., Huecas, V., & Del Campo, A. L. G. (1989). Hematology and blood chemistry of the Chilean flamingo. *Comparative Biochemistry and Physiology -- Part A: Physiology*, *94*(4), 623–625. doi:10.1016/0300-9629(89)90604-X

Puerta, M., Nava, M. P., Venero, C., & Veiga, J. P. (1995). Hematology and plasma chemistry of house sparrows (Passer domesticus) along the summer months and after testosterone treatment. *Comparative Biochemistry and Physiology -- Part A: Physiology*, *110*(4), 303–307. doi:10.1016/0300-9629(94)00187-X

Reissig, E. C., Robles, C. A., & Sager, R. (2002). Hematology and serum chemistry values of the lesser rhea (Pterocnemia pennata) raised in Patagonian farms (Argentina). *Journal of Zoo and Wildlife Medicine*, *33*(4), 328–331. doi:10.1638/1042-7260(2002)033[0328:HASCVO]2.0.CO;2

Ritchie, B. W., Hsarrison, G. J., Zantop, D., & Harrison, L. R. (1994). *Avian Medicine: Principles and Application. Hematology and biochemistry.* Lake Worth, FL: Wingers Publishing.

Rodewald, P. (Ed.). (2015). *Birds of North America Online*. Ithaca, NY: Cornell Laboratory of Ornithology. Retrieved from http://bna.birds.cornell.edu/bna

Speckmann, G. (1986). Zoo and Wild Animal Medicine. *The Canadian Veterinary Journal*. doi:10.1016/B978-1-4377-1986-4.00083-4

Van den Steen, E., Eens, M., Geens, A., Covaci, A., Darras, V. M., & Pinxten, R. (2010). Endocrine disrupting, haematological and biochemical effects of polybrominated diphenyl ethers in a terrestrial songbird, the European starling (Sturnus vulgaris). *Science of the Total Environment*, *408*(24), 6142–6147. doi:10.1016/j.scitotenv.2010.09.003

Verhulst, S., Oosterbeek, K., & Bruinzeel, L. W. (2002). Haematological parameters, mass and moult status in dunlins Calidris alpina preparing for spring migration. *Avian Science*, *2*(4), 199–206. Retrieved from http://www.eounion.org/pdf/v24/AS-2-4-Verhulst et al.pdf

Williams, J. B., & Braun, E. J. (1996). Renal compensation for cecal loss in Gambel's quail (Callipepla gambelii). *Comparative Biochemistry and Physiology - A Physiology*, *113*(4), 333–341. doi:10.1016/0300-9629(95)02073-X

Work, T. M. (1996). Weights, hematology and serum chemistry of seven species of free-ranging tropical pelagic seabirds. *Journal of Wildlife Diseases*, *32*(4), 643–657. doi:10.7589/0090-3558-32.4.643

Table S5. Data and references for haematology and energetics.

Species	Mass (g)	BMR (W)	FMR (W)	Hct (%)	AEE (W)	Energetics Ref.
Struthio_camelus	88300	60.13	216.644	45	156.514	(K . A . Nagy, Girard, & Brown, 1999; Tieleman & Williams, 2000; Williams et al., 1993; Withers & Fitzpatrick, 1983)
Alle_alle	1644.267	2.056	8.034	56.8	5.978	(Gabrielsen, Taylor, Konarzewski, & Mehlum, 1991)
Anas_rubripes	1026	3.6319	6.86	40.2	3.2281	(Wooley & Owen, 1978)
Phoebastria_immutabilis	3066	7.975	20.8681	37.9	12.8931	(Ellis & Gabrielsen, 2002)
Spheniscus_demersus	3169	8.727	22.4902	46.7	13.7632	(Kenneth . A . Nagy, Siegfried, & Wilson, 1984)
Callipepla_gambelii	143.417	0.53611	1.0516	42.4	0.51549	(Goldstein & Nagy, 1985)
Thalassarche_chrysostoma	3706.25	8.5083	28.175	41.67	19.6667	(Costa & Prince, 1987; McNab, 2009)
Tyto_alba	533.2	1.794	2.465	42.42	0.671	(McNab, 2009; Thouzeau, Duchamp, & Handrich, 1999)
Aptenodytes_patagonicus	13500	21.8528	111.1988	47.8	89.346	(Kooyman, Gentry, Bergman, & Hammel, 1976;

						McNab, 2009)
Pygoscelis_papua	5848	15.471	53.819	45.3	38.348	(K . A . Nagy et al., 1999)
Larus_dominicanus	980.3	7.06	9.259	39	2.199	(Ellis & Gabrielsen, 2002; Griffiths & Hockey, 1987)
Phalacrocorax_carbo	1950	8.345	31.725	45.4	23.38	(Barrett & Pedersen, 2001)
Ciconia_ciconia	3440	9.24	30.09	44.1	20.85	(Mata, Massemin-Challet, Caloin, Michard-Picamelot, & Maho, 2010; Pennycuick, 1971)
Pygoscelis_adeliae	3970	12.233	43.866	46.2	31.633	(K . A . Nagy et al., 1999)
Falco_tinnunculus	213	0.903	2.6	45.9	1.697	(Masman & Klaassen, 1987)
Macronectes_giganteus	3939.062 5	13.356	48.8571	43.4	35.501 1	(McNab, 2009; Kenneth . A . Nagy & Obst, 1992)
Eudyptes_chrysolophus	3870	13.75	50.694	50.35	36.944	(Ellis & Gabrielsen, 2002)
Sula_sula	1069.889	9.7	14.178	49	4.478	(Ballance, 1995)
Cacatua_roseicapilla	311.333	1.25	4.2554	48.8	3.0054	(McNab, 2009; Williams, Withers, Bradshaw, & Nagy, 1991)
Eudyptula_minor	1120.25	3.192	17.514	41.2	14.322	(McNab, 2009)

Sterna_fuscata	186	1.00694	2.78935	47	1.78241	(Ellis & Gabrielsen, 2002)
Morus_capensis	2580	8.75	39.1204	54.15	30.3704	(Ellis & Gabrielsen, 2002)
Pygoscelis_antarcticus	3515.5	10.88	64.815	50.1	53.935	(K . A . Nagy et al., 1999)
Falco_sparverius	119	0.845	2.08	52.5	1.235	(K . A . Nagy et al., 1999)
Calidris_canutus	143.75	0.8806	2.909	51.25	2.0284	(Cartar & Morrison, 1997; McNab, 2009)
Fratercula_arctica	460	3.5764	9.8148	57.9	6.2384	(Ellis & Gabrielsen, 2002)
Uria_lomvia	980	6.3889	21.5278	52.8	15.1389	(Ellis & Gabrielsen, 2002)
Turdus_merula	82.6	0.928	2.072	45	1.144	(McNab, 2009)
Uria_aalge	940	4.5139	20.703	55.3	16.1891	(Cairns, Montevecchi, Birt-Friesen, & Macko, 1990; McNab, 2009)
Fulmarus_glacialis	755.429	3.8194	16.719	44	12.8996	(Furness & Bryant, 1996; McNab, 2009)
Melopsittacus_undulatus	35	0.369	0.5969	46.8	0.2279	(Hudson, Isaac, & Reuman, 2013; McNab, 2009)
Rissa_tridactyla	373.509	2.7444	9.874	54.9	7.1296	(Fyhn, M. et al., 2001; McNab, 2009)
Ptychoramphus_aleuticus	173.989	1.6863	4.78	53	3.0937	(Hodum, Sydeman,

						Visser, & Weathers, 1998; Richman & Lovvorn, 2011)
Quiscalus_quiscula	92.2	0.989	2.8819	43	1.8929	(McNab, 2009; Peer, Homan, Linz, & Bleier, 2003)
Sterna_hirundo	130.1	1.5046	4.1157	48.1	2.6111	(Galbraith, Hatch, Nisbet, & Kunz, 2017; Klaassen, Becker, & Wagener, 1992)
Sturnus_vulgaris	73.9	0.878	2.64	57.5	1.762	(McNab, 2009)
Puffinus_pacificus	384	2.2106	7.1065	48	4.8959	(Ellis & Gabrielsen, 2002)
Sterna_paradisaea	101.125	0.8102	3.8729	46	3.0627	(Klaassen, Bech, Masman, & Slagsvold, 1989; Uttley, Tatner, & Monaghan, 1994)
Oceanites_oceanicus	42.692	0.4282	1.666	49.3	1.2378	(Obst, Nagy, & Ricklefs, 1987)
Passer_domesticus	29.3	0.367	1.157	48.25	0.79	(McNab, 2009)
Pyrrhula_pyrrhula	29.5	0.5528	1.0185	53.8	0.4657	(Bryant, 1997; McNab, 2009)
Muscicapa_striata	14.4	0.2472	0.6046	55.433	0.3574	(Bryant, 1997; McNab, 2009)

Zonotrichia_leucophrys	26.1	0.336	1.113	50.2	0.777	(McNab, 2009)
Erithacus_rubecula	16.3	0.281	0.805	48.5	0.524	(McNab, 2009)
Delichon_urbicum	20.1	0.1278	0.892	53.2	0.7642	(McNab, 2009; K . A . Nagy, 1987)
Junco_hyemalis	20.8	0.383	0.81597	50.45	0.43297	(W. . W. . Weathers & Sullivan, 1993)
Pelecanoides_urinatrix	137	1.5046	6.4468	42.4	4.9422	(Ellis & Gabrielsen, 2002)
Parus_montanus	11.5	0.275	0.5445	44.9	0.2695	(Carlson, Moreno, & Alatalo, 1993; McNab, 2009)
Malurus_cyaneus	8.25	0.1701	0.3958	42.174	0.2257	(W. . W. . Weathers & Stiles, 1989)
Ficedula_hypoleuca	14.5	0.2333	0.7237	51.89	0.4904	(McNab, 2009; J Moreno, Cowie, Sanz, & Williams, 1995)
Phylidonyris_novaehollandiae	17.2	0.6615	0.8587	49.65	0.1972	(Kusuma & Rose, 2005; W. Weathers, Paton, & Seymour, 1996)
Passerculus_sandwichensis	19.033	0.222	0.976	57.7	0.754	(McNab, 2009; Williams, 1987)
Calypte_anna	4.71	0.115	0.269	55.3	0.154	(Beuchat, Chaplin, & Morton, 1979; Lasiewski, 1963)

Hirundo_rustica	19	0.3	1.243	52.68	0.943	(McNab, 2009; K . A . Nagy et al., 1999)
Carduelis_tristis	13.5	0.336	0.75	53.5	0.414	(Buttemer, 1985; Tieleman & Williams, 2000)
Parus_gambeli	11.7	0.282	0.666	50	0.384	(Cooper, 2000)
Ficedula_albicollis	15.925	0.2757	0.9093	51.4	0.6336	(Juan . Moreno, Gustafsson, Carlson, & Part, 1991)
Melospiza_melodia	21.6	0.25	1.275	52.6	1.025	(Helms, 1963; McNab, 2009)
Parus_major	17.7	0.406	1.1	44.5	0.694	(Lasiewski & Dawson, 1967; Tinbergen & Dietz, 1994)
Parus_atricapillus	10.4	0.253	0.769	48.1	0.516	(Karasov, Brittingham, & Temple, 1992; McNab, 2009)
Zonotrichia_albicollis	24	0.278	1.681	47.2	1.403	(Helms, 1963; McNab, 2009)
Tachycineta_bicolor	17.695	0.372	1.5168	41.71	1.1448	(Burness, Hochachka, & Ydenberg, 1998; Tinbergen & Dietz, 1994)
Selasphorus_rufus	3.6	0.0675	0.322	61.1	0.2545	(Lasiewski, 1963; Schmidt-Nielsen, 1997)

Buteo_jamaicensis	1384	5.71	7.0373	46.8	1.3273	(Pakpahan, Haufler, & Prince, 1989; Soltz, 1984)
Branta_leucopsis	2100	9.104	78.5	43.1	69.396	(Butler, Woakes, & Bishop, 1998; Portugal, Green, & Butler, 2007)

References

Ballance, L. . T. . (1995). Flight energetics of free-ranging red-footed boobies (Sula sula). *Physiological Zoology*, *68*(5), 887–914.

Barrett, R. T., & Pedersen, T. (2001). Foraging strategies of great cormorants phalacrocorax carbo carbo wintering north of the arctic circle. *Bird Study*, *48*(1), 59–67. doi:10.1080/00063650109461203

Beuchat, C. ., Chaplin, S. ., & Morton, M. . (1979). Ambient temperature and the daily energetics of two species of hummingbirds, calypte anna and selasphorus rufus. *Physiological Zoology*, *52*(3), 280–295.

Bryant, D. . M. . (1997). Energy expenditure in wild birds. *Proceedings of the Nutrition Society*, *56*, 1025–1039.

Burness, G. ., Hochachka, P. ., & Ydenberg, R. . (1998). Interindividual variability in body composition and resting oxygen consumption rate in breeding tree swallows, Tachycineta bicolor. *Physiological Zoology*, *71*(3), 247–256.

Butler, P. . J. ., Woakes, A. . J. ., & Bishop, C. . M. . (1998). Behaviour and physiology of Svalbard Barnacle Geese Branta leucopsis during their autumn migration. *Journal of Avian Biology*, *29*, 536–545.

Buttemer, W. . A. . (1985). Energy relations of winter roost-site utilization by American goldfinches (Carduelis tristis). *Oecologia*, *68*, 126–132.

Cairns, D. . K. ., Montevecchi, W. . A. ., Birt-Friesen, V. . L. ., & Macko, S. . A. . (1990). Energy expenditures, activity budgets, and prey harvest of breeding Common Murres. *Studies in Avian Biology*, *14*, 84–92.

Carlson, A., Moreno, J., & Alatalo, R. V. (1993). Winter Metabolism of Coniferous Forest Tits (Paridae) Under Arctic Conditions - A Study with Doubly Labeled Water. *Ornis Scandinavica*, *24*(2), 161–164.

Cartar, R. V., & Morrison, R. I. G. (1997). Estimating metabolic costs for homeotherms from weather data and morphology: an example using calidridine sandpipers. *Canadian Journal of Zoology*, *75*(1), 94–101. doi:10.1139/z97-012

Cooper, S. . J. . (2000). Seasonal energetics of mountain chickadees and juniper titmice. *The Condor*, *102*(3), 635–644.

Costa, D. . P. ., & Prince, P. . A. . (1987). Foraging energetics of grey-headed albatrosses Diomedea chrysostoma at Bird Island, South Georgia. *Ibis*, *129*, 149–158.

Dunning Jr., J. B. (2008). *CRC Handbook of Avian Body Masses*. *CRC handbook of avian body masses. Second edition.* doi:10.1017/S0963180113000479

Ellis, H. . I. ., & Gabrielsen, G. . W. . (2002). Energetics of free-ranging seabirds. In *Biology of Marine Birds* (pp. 359–407).

Furness, R. . W. ., & Bryant, D. . M. . (1996). Effect of wind on field metabolic rates of breeding northern fulmars. *Ecology*, *77*, 1181–1188.

Fyhn, M., G., Gabrielsen, W., Nordøy, E. S., Moe, B., Langseth, I., & Bech, C. (2001). Individual variation in

field metabolic rate of kittiwake (Rissa tridactyla) during the chick rearing period. *Physiological and Biochemical Zoology*, *74*, 343–355.

Gabrielsen, G. . W. ., Taylor, J. . R. . E. ., Konarzewski, M. ., & Mehlum, F. . (1991). Field and laboratory metabolism and thermoregulation in dovekies (Alle alle). *The Auk*, *108*(January), 71–78.

Galbraith, H. ., Hatch, J. . J. ., Nisbet, I. . C. . T. ., & Kunz, T. . H. . (2017). Age-related changes in efficiency among breeding common terns Sterna hirundo: measurement of energy expenditure using doubly-labelled water. *Journal of Avian Biology*, *30*(1), 85–96.

Goldstein, D. . L. ., & Nagy, K. . A. . (1985). Resource utilization by desert quail: time and energy, food and water. *Ecology*, *66*(2), 378–387.

Griffiths, C. . L. ., & Hockey, P. . (1987). A model describing the interactive roles of predation, competition and tidal elevation in structuring mussel populations. *South African Journal of Marine Science*, *5*(1), 547–556. doi:10.2989/025776187784522496

Helms, C. . W. . (1963). Tentative field estimates of metabolism in buntings. *The Auk*, *80*(3), 318–334.

Hodum, P. . J. ., Sydeman, W. . J. ., Visser, H. ., & Weathers, W. . W. . (1998). Energy expenditure and food requirements of Cassin's auklets provisioning nestlings. *Condor*, *100*, 546–550.

Hudson, L. . N. ., Isaac, N. . J. . B. ., & Reuman, D. . C. . (2013). The relationship between body mass and field metabolic rate among individual birds and mammals. *Journal of Animal Ecology*, *82*(1), 1009–1020. doi:10.1111/1365-2656.12086

Karasov, W. . H. ., Brittingham, M. . C. ., & Temple, S. . A. . (1992). Daily energy and expenditure by black-capped chickadees (Parus atricapillus) in winter. *The Auk*, *109*(2), 393–395.

Klaassen, M. ., Bech, C. ., Masman, D. ., & Slagsvold, G. . (1989). Growth and Energetics of Arctic tern chicks (Sterna paradisaea). *The Auk*, *106*(2), 240–248.

Klaassen, M. ., Becker, P. . H. ., & Wagener, M. . (1992). Transmitter loads do not affect the daily energy expenditure of nestling common terns. *Journal of Field Ornithology*, *63*, 181–185.

Kooyman, G. . L. ., Gentry, R. . L. ., Bergman, W. . P. ., & Hammel, H. . T. . (1976). Heat Loss in penguins during immersion and compression. *Comparative Biochemistry and Physiology*, *54A*, 75–80.

Kusuma, L. . P. . E. ., & Rose, R. . W. . (2005). Metabolism of winter-acclimatized New Holland Honeyeaters Phylidonyris novaehollandiae from Hobart, Tasmania. *Acta Zoologica Sinica*, *51*(2), 338–343.

Lasiewski, R. . C. . (1963). Oxygen consumption of torpid, resting, active, and flying hummingbirds. *Physiological Zoology*, *36*(2), 122–140.

Lasiewski, R. . C. ., & Dawson, W. . R. . (1967). A re-examination of the relation between standard metabolic rate and body weight in birds. *The Condor*, *69*(1), 13–23.

Masman, D. ., & Klaassen, M. . (1987). Energy expenditure during free flight in trained and free-living Eurasian kestrels (Falco Tinnunculus), *104*(October), 603–616.

Mata, A. ., Massemin-Challet, S. ., Caloin, M. ., Michard-Picamelot, D. ., & Maho, Y. . L. . (2010). Seasonal

variation in energy expenditure and body composition in captive White Storks (Ciconia ciconia). *Comparative Biochemistry and Physiology, Part A*, *155*(1), 19–24. doi:10.1016/j.cbpa.2009.08.019

McNab, B. . K. . (2009). Ecological factors affect the level and scaling of avian BMR. *Comparative Biochemistry and Physiology, Part A*, *152*(1), 22–45. doi:10.1016/j.cbpa.2008.08.021

Moreno, J. ., Gustafsson, L. ., Carlson, A. ., & Part, T. . (1991). The cost of incubation in relation to clutch-size in the collared flycatcher Ficedula albicdis. *Ibis*, *133*, 186–192.

Moreno, J., Cowie, R., Sanz, J., & Williams, R. (1995). Differential response by males and females to brood manipulations in the Pied Flycatcher: Energy expenditure and nestling diet. *Journal of Animal Ecology*, *64*(6), 721–732. doi:10.2307/5851

Nagy, K. . A. . (1987). Field metabolic rate and food requirement scaling in mammals and birds. *Ecological Monographs*, *57*(2), 111–128.

Nagy, K. . A. ., Girard, I. . A. ., & Brown, T. . K. . (1999). Energetics of free-ranging mammals, reptiles, and birds. *Annual Review of Nutrition*, *19*(1), 247. doi:https://doi.org/10.1146/annurev.nutr.19.1.247

Nagy, K. . A. ., & Obst, B. . S. . (1992). Food and energy requirements of Adelie penguins (Pygoscelis adellae) on the Antarctic Peninsula. *Physiological Zoology*, *65*(6), 1271–1284.

Nagy, K. . A. ., Siegfried, W. . R. ., & Wilson, R. . P. . (1984). Energy utilization by free-ranging jackass penguins, Spheniscus demersus. *Ecology*, *65*(5), 1648–1655.

Obst, B. . S. ., Nagy, K. . A. ., & Ricklefs, R. . E. . (1987). Energy utilization by Wilson's storm-petrel (Oceanites oceanicus). *Physiological Zoology*, *60*(2), 200–210.

Pakpahan, A. . M. ., Haufler, J. . B. ., & Prince, H. . H. . (1989). Metabolic rates of red-tailed hawks and great horned owls. *The Condor*, *91*(April), 1000–1002.

Peer, B. . D. ., Homan, H. . J. ., Linz, G. . M. ., & Bleier, W. . J. . (2003). Impact of blackbird damage to sunflower: Bioenergetic and economic models. *Ecological Applications*, *13*(1), 248–256. doi:10.1890/1051-0761(2003)013[0248:IOBDTS]2.0.CO;2

Pennycuick, C. . J. . (1971). Soaring behaviour and performance of some East African birds, observed from a motor-glider. *Ibis*, *114*, 178–218.

Portugal, S. . J. ., Green, J. . A. ., & Butler, P. . J. . (2007). Annual changes in body mass and resting metabolism in captive barnacle geese (Branta leucopsis): the importance of wing moult. *The Journal of Experimental Biology*, *210*, 1391–1397. doi:10.1242/jeb.004598

Richman, S. . E. ., & Lovvorn, J. . R. . (2011). Effects of Air and Water Temperatures on Resting Metabolism of Auklets and Other Diving Birds. *Physiological and Biochemical Zoology*, *84*(3), 316–332. doi:10.1086/660008

Schmidt-Nielsen, K. (1997). *Animal Physiology* (5th ed.). Cambridge: Cambridge University Press.

Soltz, R. . L. . (1984). Time and energy budgets of the red-tailed hawk in Southern California. *The Southwestern Naturalist*, *29*(2), 149–156.

Thouzeau, C. ., Duchamp, C. ., & Handrich, Y. . (1999). Energy metabolism and body temperature of barn

owls fasting in the cold. *Physiological and Biochemical Zoology*, *72*(2), 170–178.

Tieleman, B. . I. ., & Williams, J. . B. . (2000). The adjustment of avian metabolic rates and water fluxes to desert environments. *Physiological and Biochemical Zoology*, *73*(4), 461–479.

Tinbergen, J. M., & Dietz, M. W. (1994). Parental energy expenditure during brood rearing in the great tit (Parus major) in relation to body mass, temperature, food availability and clutch size. *Functional Ecology*, *8*(5), 563–572. doi:10.2307/2389916

Uttley, J. ., Tatner, P. ., & Monaghan, P. . (1994). Measuring the daily energy expenditure of free -living Arctic terns (Sterna paradisaea). *The Auk*, *111*(2), 453–459.

Weathers, W. . W. ., & Stiles, F. . G. . (1989). Energetics and water balance in free -living tropical hummingbirds. *The Condor*, *91*(2), 324–331.

Weathers, W. . W. ., & Sullivan, K. . A. . (1993). Seasonal patterns of time and energy allocation by birds. *Physiological Zoology*, *66*(4), 511–536.

Weathers, W., Paton, D., & Seymour, R. (1996). Field Metabolic Rate and Water Flux of Nectarivorous Honeyeaters. *Australian Journal of Zoology*, *44*(5), 445. doi:10.1071/ZO9960445

Williams, J. . B. . (1987). Field metabolism and food consumption of savannah sparrows during the breeding season. *The Auk*, *104*(2), 277–289.

Williams, J. . B. ., Siegfried, W. . R. ., Milton, S. . J. ., Adams, N. . J. ., Dean, W. . R. . J. ., du Plessis, M. . A. ., & Jackson, S. . (1993). Field metabolism, water requirements, and foraging behavior of wild ostriches in the Namib Dean. *Ecology*, *74*(2), 390–404.

Williams, J. B., Withers, P. C., Bradshaw, S. D., & Nagy, K. A. (1991). Metabolism and water flux of captive and free-living Australian parrots. *Australian Journal of Zoology*, *39*(2), 131–142. doi:10.1071/ZO9910131

Withers, P. . C. ., & Fitzpatrick, P. . (1983). Energy, water, and solute balance of the ostrich Struthio camelus. *Physiological Zoology*, *56*(4), 568–579.

Wooley, J. . B. ., & Owen, R. . B. . (1978). Energy costs of activity and daily energy expenditure in the black duck. *The Journal of Wildlife Management*, *42*(4), 739–745.

Figure S6. Relationship between A) Hct and sampling season, B) Hb and sampling season.

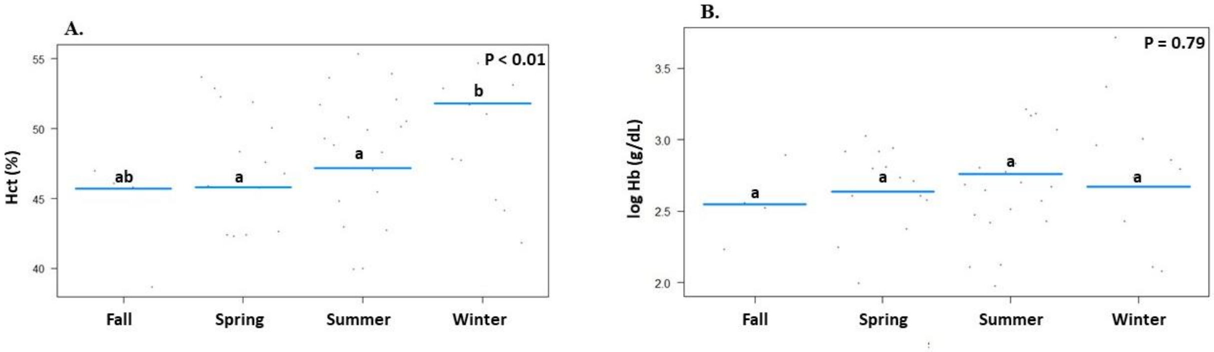

Supplement: Supplementary file 1 — Supplementary information and dataset [file 41598_2019_42921_MOESM1_ESM.pdf]
